# Quantification of trace ^227^Ac and other radionuclidic impurities in mass-separated ^225^Ac samples produced at CERN-MEDICIS

**DOI:** 10.1038/s41598-025-02277-4

**Published:** 2025-07-02

**Authors:** Jake D. Johnson, Cyril Bernerd, Frank Bruchertseifer, Thomas E. Cocolios, Marie Deseyn, Charlotte Duchemin, Michael Heines, Max Keppens, Laura Lambert, Nathan Meurrens, Ralf E. Rossel, Thierry Stora, Viktor Van den Bergh

**Affiliations:** 1 KU Leuven, IKS, Leuven, Belgium; 2 JRC, Karlsruhe, Germany; 3 CERN, Geneva, Switzerland

**Keywords:** Experimental nuclear physics, Mass spectrometry

## Abstract

^225^Ac is a promising candidate medical radionuclide for targeted alpha therapy of advanced stage cancers. One of the main production pathways is the high-energy proton spallation of thorium-based targets, that requires an efficient, nuclide-selective separation method to recover ^225^Ac from hundreds of co-produced spallation and fission products. The main radioactive contaminant of concern is ^227^Ac  (T_1/2_ = 21.8 years), that could preclude extensive medical use if not significantly suppressed. In this work, ^225^Ac samples were produced by mass separation of radioactive ion beams extracted from proton-irradiated thorium-based targets. The activity of ^225^Ac and other possible contaminants of the samples were measured using complementary gamma- and alpha-decay spectrometry methods, while ^227^Ac activity was calculated by performing alpha-decay spectrometry of recoiled progeny from the sample. Using this novel method, accurate measurement of trace ^227^Ac activity in ^225^Ac samples was performed much faster than with conventional spectrometry techniques, thanks to its 10,000-fold increase in relative sensitivity. The end of collection activity ratio of ^227^Ac to ^225^Ac in two samples from irradiated targets were determined to be $2.00(10) \times 10^{-6}$ and $2.7(4) \times 10^{-6}$ respectively, three orders of magnitude below the ^227^Ac activity in ^225^Ac products obtained through radiochemical separation. The high separation factor of ^225^Ac over ^227^Ac suggests the suitability of mass-separated accelerator-based ^225^Ac for medical use.

## Introduction

Targeted alpha therapy (TAT) is a treatment modality of advanced stage cancers with distributed lesions that exploits the high yet selective cyto-toxicity of alpha radiation. In most cases, an alpha-emitting nuclide is chelated to a targeting molecule that is chosen for its preferential binding to tumor-specific receptors. The alpha radiation that is emitted by the nuclide in the vicinity of the targeted cancer cells causes damage through a number of mechanisms that have been described in detail^1–9^, but still require further investigation. One of the main issues limiting pre-clinical research progress is the procurement of appropriate alpha-emitting nuclides^10^. Though many alpha emitters exist, only a few are suitable for TAT due to the necessity of matching their nuclear decay half-life with the circulation times of conjugate targeting vectors. Of the suitable candidate alpha-emitting nuclides, ^225^Ac is one of the most promising due to its 9.92 day half-life^11^ and 4 rapid subsequent $\alpha$-decays, whose energy totals 28 MeV. The growing demand for medical grade ^225^Ac exceeds the yearly 70 GBq production capacity of existing ^229^Th generators, prompting the development of nuclear-reaction-based production approaches^10,12^.

High-energy proton spallation of naturally-occurring ^232^Th-based targets is a promising alternative production route, which, unlike generator production, has the potential to be scaled up. For example, at the Los Alamos National Laboratory (LANL) material test station, the ^225^Ac in-target production rate is estimated at $17~{\hbox {MBq}/{\upmu \hbox {Ad}}/{\hbox {gcm}^{-2}}}$ using a measured reaction cross section of 14.8(11) mb^13^. The end of irradiation ^225^Ac yield scales up with proton charge on target and target thickness. The monthly production yield across selected North American accelerator complexes LANL, Brookhaven National Laboratory (BNL) and TRIUMF for nominal operating conditions has been estimated to be greater than 3000 GBq/month^12^. Despite the promising production rates, a major hurdle in producing medical grade ^225^Ac through this reaction is the co-production of ^227^Ac that cannot be separated from ^225^Ac in standard radiochemical treatments of irradiated targets^14–17^. Cumulative reaction cross sections for protons on ^232^Th have been measured for both ^225^Ac and ^227^Ac at several energies of up 200 MeV^18,19^ and at higher energies of 438 MeV^14^, 800 MeV^13^ and 8 GeV^20^. The cross section of both nuclides is greater than 10 mb for proton energies above 128 MeV, with that of ^227^Ac slightly larger than that of ^225^Ac. The relative in-target activity of ^227^Ac compared to ^225^Ac produced with $\ge 70$ MeV proton beams is thus predicted to be approximately the ratio of their decay constants: the order of 0.1%. The ^227^Ac to ^225^Ac activity ratio has been measured to be 0.15(4)% and 0.142(5)% at end of irradiation in samples of thorium metal irradiated with 438 MeV and 100 MeV protons at TRIUMF (Canada)^14^ and at Brookhaven National Laboratory (USA)^21^, respectively. This ^227^Ac activity fraction raises concerns from a pharmaceutical waste management perspective^22^ and a regulatory perspective, that may impact wide-scale clinical adoption. For example, in the USA, financial assurance is required for licenses to handle $> 370$ kBq ^227^Ac, that can be burdensome for dedicated ^225^Ac production laboratories. Additionally, low limits for contamination reporting (74 Bq ^227^Ac) and accidental ingestion (15 Bq ^227^Ac) mean that some laboratories may be reluctant to handle ^225^Ac products with excessive ^227^Ac content^23^. In Switzerland, the authorization limit (LA) for ^227^Ac is 8 Bq^24^. Class C laboratories can therefore handle up to 800 Bq ^227^Ac. A limit on acceptable ^227^Ac activity in medical-grade ^225^Ac products is suggested in refs.^14^ and^25^ to be 0.01 % of ^225^Ac activity, corresponding to 1 kBq ^227^Ac per 10 MBq patient dose, in line with the exemption limit for ^227^Ac given by the International Atomic Energy Agency (IAEA)^26^. While a consensus acceptable limit of ^227^Ac activity in medical ^225^Ac products is yet to be established, the stringent limits imposed on ^227^Ac handling by regulatory authorities motivate the need to suppress it as much as possible, that in turn motivates the need of an isotopically-selective separation method of ^225^Ac from thorium-based targets irradiated with high-energy protons.

Isotope separation of reaction products from irradiated targets can be achieved through the isotope separation on line (ISOL) technique, employed for the separation and study of exotic nuclides, for example at the CERN-ISOLDE facility^27,28^. An offline adaptation of this method can be applied for batch extraction of medical radionuclides from pre-irradiated targets, as developed at the CERN-MEDICIS facility since 2018^29^. Here, element selectivity is achieved through in-source step-wise resonance laser ionization, that increases the ionization efficiency of a chosen element above the surface ionization efficiency from a hot cavity. Mass selectivity is then achieved based on the specific magnetic rigidity of the produced ion beam in the double-focusing separator magnet that is transmitted through slits. This combination provides a high degree of selectivity for the chosen nuclide over all other reactions products with the exception of volatile, efficiently surface-ionized isobaric contaminants. Previous work has shown that ^225^Ac can be ionized with a 15% efficiency using a combination of two resonance laser ionization schemes in addition to surface ionization^30^, however extraction efficiency and selectivity for ^225^Ac from irradiated thick targets is yet to be determined. In this work, the isotope selectivity of mass separation is studied by accurately quantifying the ^227^Ac content with nuclear decay spectrometry in three samples of ^225^Ac separated by mass as radioactive ion beams under different conditions. The separation enhancement factor, $f_{sep}$ is defined in eq. (1) as the relative collected activity at end of collection, $A_{e.o.c}$ to in-target activity, $A_{targ}$ for the nuclide of interest X, with respect to contaminant nuclide, Y. It has been calculated for ^225^Ac with respect to several contaminants identified with decay spectroscopy, including ^227^Ac.

1 $$\begin{aligned} f_{sep} = \frac{A_{e.o.c}(X)}{A_{targ}(X)}/\frac{A_{e.o.c}(Y)}{A_{targ}(Y)} \end{aligned}$$

In section 2, details of the Ac-source/target preparation are provided, including irradiation conditions and inventories of relevant radionuclides of the sources at the start of collection. Section 3 then describes how species from the target are ionized, separated and collected at CERN-MEDICIS, producing the 3 samples analyzed in the rest of the work. The novel method of alpha-decay recoil spectrometry for trace analysis is described in section 4, where it is applied to determine the ^227^Ac content of the samples. The activity measurements of ^225^Ac, ^225^Ra and other radioactive impurities in each of the samples using complementary decay spectrometry techniques performed at KU Leuven are presented in section 5. Finally, the resulting separation enhancement factors of ^225^Ac relative to ^227^Ac, along with the radioisotopic and radionuclidic purity of a collected sample, are discussed.

## Production of ^225^Ac-containing sources for separation

The mass-separated ^225^Ac samples measured in this work were extracted from three different sources at CERN-MEDICIS. In preparation for loading of the nuclear material, target units, developed from those used at CERN-ISOLDE, consisting of a Ta target container coupled via a Ta transfer line to a tubular Re ion source were conditioned. For this, each target unit was gradually heated under vacuum at the ISOLDE offline 1 mass separator to release typical impurities such as Na, K, Al, La and LaO, identified as ion beams produced in the Re surface ion source.

### Source A: ^227^Ac and ^225^Ac loaded on thick ThO_2_target prior to mass separation

A first source, source A, was produced in order to test the performance of the separator for a known initial quantity of ^227^Ac and ^225^Ac. This source was not an irradiated target. It was produced at JRC Karlsruhe by depositing and evaporating a mixture of 12.7  MBq ^225^Ac(NO_3_)_3_ and 100  kBq ^227^Ac(NO_3_)_3_ solutions respectively onto a fibrous ThO_2_  target of 15.08 g. The source thus constitutes the dried Ac precipitates adsorbed onto the ThO_2_  target surface. The release of Ac from this target upon heating is governed only by surface desorption and macroscopic effusion to the ion source, while Ac released from an irradiated target undergoes the additional processes of diffusion and microscopic effusion through the target bulk.

The initial activities of the nuclides were measured with alpha- and gamma-decay spectrometry for ^225^Ac, and gamma-decay spectrometry for ^227^Ac, before deposition of the solutions. The spiked ThO_2_  target was transported to the CERN-MEDICIS facility, where it was placed into one of the conditioned ISOLDE target containers. The target unit was then coupled to the front end of the mass separator. The activities of ^225^Ac and ^227^Ac at the start of collection were 10.8 MBq and 100 kBq respectively.

### Sources B and C: Irradiated Th-based targets

For source B, a sintered ThO_2_  target with a mass of 14.65 g was used. It was inserted into an ISOLDE target unit, then irradiated under Ar atmosphere at the MEDICIS irradiation station for a total time of 30 hours, with a total of $1.46 \times 10^{17}$ protons of 1.4 GeV from the proton synchrotron booster (PSB) impinging the target in the direct irradiation configuration^31^.

For source C, a thicker 98.9 g ThC_x_target was used. It was produced by reacting pressed ThO_2_  powder with excess graphite pellets in a carbothermal reduction reaction to form mostly ThC_2_ + C_2_. In preparation for the collection, the ThC_x_  target material was inserted into the target container, then characterized and conditioned by heating it on the front-end of the MEDICIS separator, as done at the ISOLDE offline 1 separator for empty target units. During the characterization process, contaminants present in the ThC_x_  target material that may contribute to background ion current at mass-to-charge ratio (*A*/*q*) = 225 were identified. Beams at $A/q =$ 224, 226, and 228 attributed to ^224, 226, 228^Ra^+^ that are produced radiogenically in the decay chains of the target material were successfully outgassed. This was done to ensure that the ion beams produced around the $A/q = 225$ region during nuclide collection from the irradiated target would only be those produced through reactions induced by the proton irradiation. At the end of the conditioning period with the target temperature at 2138 $^\circ$C, a background beam current of 0.25 pA at $A/q = 225$ was present, attributed to mass-tailing from intense lanthanide beams. Further details of the conditioning are provided in the supplementary material. After the target was outgassed and characterized, it was irradiated under Ar atmosphere for 21.85 hours with 1.4 GeV protons at an average beam current of $1.91\,\upmu \hbox {A}$, corresponding to a total number of $9.39 \times 10^{17}$ protons. The protons were incident on the target vessel in the direct irradiation configuration.

The radionuclide inventories at the start of collection from both sources B and C were calculated for the exact irradiation conditions by Monte Carlo simulation with FLUKA (fluka.cern v4-2.2 and v4-3.0, respectively)^32–34^. Reactions induced by secondary particles generated through the interaction of the proton beam with the aluminium vessel and Ta target container were accounted for. The fragmentation process was modelled accounting for coalescence of nucleons and fragment evaporation. For source B, the proton beam was modelled as a 1.26 cm full-width-half-maximum (FWHM) and 1.1 cm FWHM Gaussian beam proton in the two orthogonal transverse axes. The target was modelled as a cylinder of length 1.27 cm and radius 0.615 cm with standard ThO_2_  density of $9.7\,\hbox {g}\,\hbox {cm}^{-3}$ corresponding to a thickness of $12.3\,{\hbox {g}\,\hbox {cm}^{-2}}$ as an approximation to the true target geometry. The target then underwent a 65.5 h cooling time. The irradiation of the ThC_x_  target for source C was simulated with a 1.4 GeV Gaussian proton beam size of 0.82 cm FWHM along each transverse axis on the target of density $3.3\,{\hbox {g}\,\hbox {cm}^{-3}}$ and thickness $32\,{\hbox {g}\,\hbox {cm}^{-2}}$, with a 3.5 hour cooling time after end of beam. The simulated activity ratio of ^227^Ac to ^225^Ac at the start of collection was 0.198(9)% and 0.159(6)% for source B and C respectively. An overview of other relevant nuclides with half-lives > 1 d produced in the target that could exist as singly ionized atomic or molecular species with *A*/*q* in the range 222-228 is shown in table 1.

**Table 1:** In-target radionuclide inventories of species with beams in the $222 \le A/q \le 228$ region at beginning of collection for each of the three sources in this work.

A/q	Nuclide	Beam	Half-life	In-target activity		
				A	B	C
222	^206^Po	^206^Po^16^O^+^	8.8(1) d	0	5.82(9) MBq	2.70(5) GBq
223	^223^Ra	^223^Ra^+^	11.43(5) d	0	960 (40) kBq	310(20) MBq
224	^208^Po	^208^Po^16^O^+^	2.898(2) Y	0	55(2) kBq	20.4(3) MBq
	^224^Ra	^224^Ra^+^	3.6316(23) d	0	4.6(2) MBq	2.79(8) GBq
225	^209^Po	^209^Po^16^O^+^	124(3) Y	0	1.74(5) kBq	568(9) kBq
	^225^Ra	^225^Ra^+^	14.9(2) d	0	380(30) kBq	149(4) MBq
	^225^Ac	^225^Ac^+^	9.920(3) d	10.8(6) MBq	2.37(7) MBq	1.03(3) GBq
226	^210^Po	^210^Po^16^O^+^	138.376(2) d	0	365(8) kBq	95(2) MBq
	^226^Ra	^226^Ra^+^	1600(7) Y	0	15.8(7) Bq	4.4(2) kBq
	^226^Ac	^226^Ac^+^	29.37(12) h	0	3.12(11) MBq	5.5(2) GBq
227	^227^Ac	^227^Ac^+^	21.772(3) Y	100(5) kBq	4.7(2) kBq	1.64(2) MBq
228	^228^Ra	^228^Ra^+^	5.75(3) Y	0	1.49(14) kBq	549(39) kBq
	^228^Ac	^228^Ac^+^	6.15(2) h	0	16.7(5) kBq	9.4(2) GBq

Several isotopes of Ra are well produced over the full mass range. Amongst the Ac isotopes, in addition to ^225^Ac and the aforementioned ^227^Ac, ^226^Ac (T_1/2_ = 29.4 h) is produced with similar cross section. This is typically not considered to be problematic as it decays quickly compared to ^225^Ac. Finally, several long-lived Po isotopes are included. They may exist as ions in the $222 \le A/q \le 228$ region as singly-ionized oxide sidebands. Section 3 further discusses how these beams could be produced.

## Collection of ^225^Ac using resonance laser ionization and mass separation

Offline isotope separation with resonance laser ionization of ^225^Ac was performed on each of the three sources at CERN-MEDICIS. The details of the laser laboratory (MELISSA), beamline and mass separator are published elsewhere^35,36^. The method of mass separation and resonance laser ionization implemented at CERN-MEDICIS has been described in previous work^30^, as well as a review article of CERN-MEDICIS operations^29^, and will be presented here briefly. After irradiation, the target and ion source unit is mounted on the front end of the separator beamline. There, it is pumped until the pressure reaches $10^{-5}~{\hbox {mbar}}$. A nominal high voltage of 60 kV is then applied between the ion source and the extraction electrode. The target container and ion source are subsequently independently resistively heated to temperatures necessary for release of the most volatile elements and efficient hot cavity ionization (approximately 1600 $^\circ$C and 2200 $^\circ$C respectively). This allows the target and ion source to be effectively ‘outgassed’, allowing for the removal of both volatile reaction products and volatile impurities from the target and ion source material that can impact the ion source performance. After the outgassing period, the target container is gradually heated further to promote release of the radionuclides to the tubular ion source. At the same time, temporally- and spatially-overlapped pulsed lasers with energies of the order of 0.1 mJ/pulse are shone into the tubular ion source for efficient step-wise resonance ionization of the isotope of interest - in this case ^225^Ac. Atoms of elements that are not selectively ionized by the resonant photon field may still be surface ionized within the hot cavity. The ionized species are extracted as an ion beam and then separated by a sector dipole magnet. The beam of the mass of interest is finally implanted into a collection foil. In this work, each sample was produced by ion beam implantation for a fraction of the duration of the collection, as up to 2 other samples were collected for distribution to other laboratories. Consequently, the activities of nuclides on the samples are less than those from the entire collection, and discussion of collection efficiencies is beyond the scope of this work.

### Ionization

Species released from the target container into the ion source enter the hot cavity environment. Even though Ac-selective ionizing lasers are shone into the cavity, all elements are ionized with an efficiency, $\epsilon _s$ given by the Saha-Langmuir equation. The equation is modified to account for ion confinement in the hot cavity therein with a temperature-dependent amplification factor, $\kappa (T)$, that depends on the partial pressures of elements in the cavity, in addition to the cavity geometry, cavity surface material and its rate of thermionic emission^37–39^.

2 $$\begin{aligned} \epsilon _s = \frac{\kappa (T) \exp \left( \frac{\phi - IP_{eff}}{k_B T}\right) }{1+ \kappa (T) \exp \left( \frac{\phi - IP_{eff}}{k_B T}\right) } \end{aligned}$$

Here, $\phi$ is the work function of the ion source material, $k_B$ is Boltzmann’s constant, *T* is the ion source temperature and $IP_{eff}$ is the effective first ionization potential of the element considered that is related to its ionization potential, $IP_{ion}$, and ion and ground state multiplicities, $g_+$ and $g_0$ respectively, by $IP_{eff} = IP_{ion} - k_B T \ln (g_+/g_0)$.

Of the species shown in table 1, the ionization potentials of Ra and Ac are known while that of PoO is not. The amplification factor, $\kappa$, is challenging to quantify due to, amongst other factors, lack of information on the partial pressure of released reaction products and volatile impurities evaporated from the target container and tubular ion source. Nonetheless, over a broad illustrative range of $1< \kappa < 50$, the surface ionization efficiency of Ra and Ac in a rhenium cavity at 2100 $^\circ$C varies from 29 % to 95 % and 3 % to 60 % respectively. Ra is thus more efficiently surface ionized than Ac by a factor ranging from 10 to 1.5. Little information is available on PoO, however it could be formed either by ionization of PoO, or through dissociation of a PoO_X_ or PoOX molecule. The relatively low surface ionization efficiency of ^225^Ac for typical ion source amplification factors, is why resonance laser ionization is used.

The resonance laser ionization on ^225^Ac was performed using two 2-step laser schemes used in parallel, shown in Fig. 2 in ref.^30^. These schemes, developed by Raeder et al^40^, and Ferrer et al^41^, share a common first step excitation and thus require three lasers. A grating Ti:Sa laser was used for generating the first step wavelength of 438.575 nm, while two birefringent filter/Fabry-Perot etalon Ti:Sa lasers were each tuned to produce 1 of the 2 second-step wavelengths of 456.148 nm and 424.702 nm. For the collection of the samples A, B and C, powers of 65, 330 and 350 mW were used respectively for the first step, while powers of between 750-960 mW were attained for the two second steps. During collections at MEDICIS, the saturation of the transitions of each step in the composite scheme was measured. It was found that the first step was saturated above powers of the order 150 mW. Step 2a was saturated above powers on the order 600 mW, while step 2b was not observed to be saturated at the laser powers achieved for all collections. The increase in beam current when the laser light was shone into the source (laser ON/OFF enhancement) was in each case used as a tool to identify ^225^Ac^+^.

### Mass separation

The mass separation step separates the beams according to the square root of their mass to charge ratio. The ability to separate beams of neighboring masses in space is quantified as the mass resolving power, *mrp*, given by

3 $$\begin{aligned} mrp = \frac{dM}{\Delta d}, \end{aligned}$$

where *d* is the distance at the separator focal plane between the chosen beam of mass, *M*, and the beam of mass $M+1$, and $\Delta d$ is the spatial full-width at half-maximum (FWHM) of the beam of mass M in the focal plane. The MEDICIS separator operating in nominal conditions reaches $mrp \approx 400$^36^. Since ^225^Ac is a relatively high-mass isotope, the relative distance to neighboring peaks in units of the beam FWHM is only 1.8, so cross-contamination from neighboring mass beams may be possible. A simulation was performed using python to calculate the beam centroid positions at the focal plane of the mass separator for the MEDICIS separator characteristics given in ref.^36^, assuming a uniform magnetic field in the separator region. Separation enhancement factors, $f_{sep}$, for $M = 225$ with respect to neighboring and next-to-neighboring masses with Gaussian beam shapes of FWHM given by eq. (3) for a range of mrp values were then calculated. This was done by Monte-Carlo counting of ions transmitted through slits of 3 mm opening distance and a circular colimator of radius, $r_c = 5$ mm, corresponding to the experimental configuration for collection from source C. The simulated separation enhancement factor over neighboring masses increased exponentially with mrp, and values of $f_{sep} = 62$ and $f_{sep} = 450$ were calculated for $mrp = 300$ and $mrp = 400$ respectively. Therefore it is expected that ions of $A/q = 224$ and $A/q = 226$ should be suppressed by approximately 2 orders of magnitude, with a precise value that depends heavily on the mrp and other optical aberrations. Separation enhancement factors over next-to-neighboring masses of $f_{sep} = 1.2 \times 10^5$ and $f_{sep} = 7.3 \times 10^6$ were obtained for $mrp = 300$ and $mrp = 400$ respectively. The values are much larger than for the neighboring-mass case due to rapid decay of Gaussian tails. The simulated separation enhancement factors are likely overestimated as scattering of ions from residual gas atoms in the separator or drift chamber, as well as other optical corrections, lead to non-Gaussian tailing^42–44^. Consequently, it is expected that both ^226^Ac and ^227^Ac are co-implanted with ^225^Ac, albeit with high suppression factors. A negligible fraction of ions that are more than two mass-to-charge units away from $A/q = 225$ is expected to be implanted.

### Sample collection conditions

The collection of sample A was performed at an extraction voltage of 50 kV, to avoid too frequent high-voltage discharges. Samples B and C were collected with an extraction voltage of 60 kV. In each case, ^225^Ac was identified through laser ON/OFF enhancement and confirmation of resonances at the frequencies of the known ionization schemes^40,41^. The target container temperatures at which ^225^Ac was first observed were 2160 °C, 2260 °C and 2260 °C for collections of samples A, B and C respectively. Sample A was collected at high target container temperatures from 2160 °C to 2430 °C. During the collection of this sample, slit position optimization and separator magnetic field optimization were performed that could have impacted the transmitted beam purity. The collection of sample B proceeded before ^225^Ac was identified with laser ON/OFF enhancement. The sample was first implanted with ^225^Ra^+^ that is released at lower target temperature, before ^225^Ac^+^ was implanted at higher temperatures. In preparation for collection on sample C, Ra^+^ was first identified in the beam due to its characteristic isotopic composition evaluated through a mass scan (shown in supplementary material). The ^225^Ra was collected on a different sample foil until the $A/q =225$ beam current had diminished by a factor 140 from 700 pA to 5 pA that did not increase with further heating. ^225^Ac^+^ was subsequently identified through the laser ON/OFF enhancement once the target was heated further. Two implantations, each of 30 m duration at 31 and 47 hours since start of collection were performed on sample C. Additional details of all collections are included in the supplementary material. Typical ion beam currents measured on the foils during the ^225^Ac sample collections were of the order of 1 pA to 10 pA.

Following the respective collections, samples A, B and C were sent to KU Leuven where an extensive decay spectrometry campaign was run to fully analyse the activities of nuclides on the sample foils at end of collection (e.o.c.).

## ^227^Ac activity determined with alpha-decay spectrometry of recoil progeny

Direct nuclear-decay spectrometry of the sample to evaluate decay rates of nuclides in the ^227^Ac chain was challenging, as any signal was estimated to be below the minimum detectable activity (MDA) due to high background radiation from nuclides in the ^225^Ac decay chain. To tackle this limitation, the novel method of using alpha-decay spectrometry of recoil progeny ($\alpha$-srp) for trace analysis was developed. The technique was applied to determine the ^227^Ac activity before waiting for ^225^Ac to decay over several half lives to reduce background and increase the MDA for ^227^Ac detection through its decay progeny. The method is based on alpha-decay spectrometry of progeny that are ejected from the sample foil onto a Si detector by virtue of their alpha-decay recoil energy that is larger than the ion beam implantation energy. This well-known phenomenon is often considered a nuisance for alpha-decay spectrometry, prompting many studies to consider how to suppress recoiling progeny from contaminating detectors^45–47^. The alpha-decay recoil has been frequently exploited at the JRC for producing sources of alpha recoil progeny for precise half-life and other nuclear data measurements^48–51^. Herein is the first time the phenomenon is exploited as a trace analysis technique to the authors’ knowledge.

**Fig. 1:**
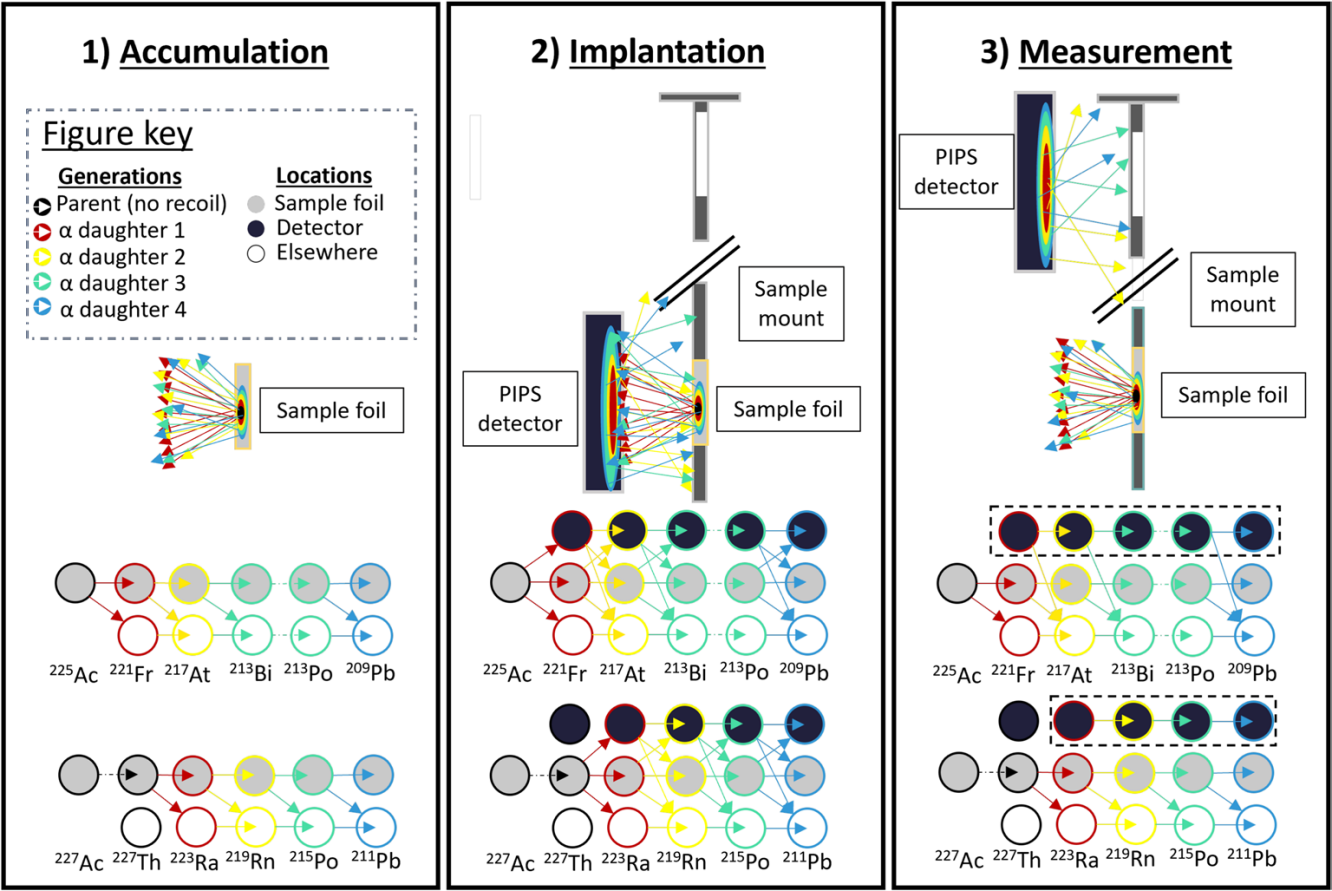
Schematic overview of the stages of alpha recoil decay spectrometry. In each stage, the dynamics of recoiling daughters are illustrated using the colors described in the key. The decay schematic for both ^227^Ac and ^225^Ac is shown at the bottom of each panel, indicating the possible transitions between states composed of the product of isotope generation and location. The dashed box in the ‘measurement’ panel shows the nuclide chains that are measured through this method.

### Methods

Figure 1 shows a schematic overview of the $\alpha$-srp technique. In the accumulation phase, ^225^Ac daughters reach secular equilibrium on the order of a few hours. Even if the sample contains ^225^Ra, the activity of ^225^Ac progeny differ from their secular equilibrium values by less than 1% after 1 day. Meanwhile, ^227^Ac progeny grow-in towards secular equilibrium that is reached on the order of 100 days for ^223^Ra and its short-lived decay progeny. After this accumulation time, $t_{acc}$, the sample is placed in front of a $300~{\hbox {mm}^{2}}$ passivated implanted planar silicon (PIPS) detector in a vacuum chamber where alpha-decay recoil progeny are implanted into the detector during a time, $t_{imp}$. After implantation, the sample mount is moved laterally by 19.5 cm such that the geometric efficiency for alpha emissions from nuclei in the sample is 0 thanks to the 1 mm recess of the detector active area behind its casing. The residual activity on the detector is then measured for a time, $t_{meas}$.

The dotted box in the measurement panel in Fig. 1 shows how this technique allows the measurement of only the decay chains of the first alpha-decay daughter of ^227^Ac and ^225^Ac as they are no longer populated by decays from the sample foil. All implanted ^225^Ac alpha-decay recoil progeny decay within the first day of measurement due to the relatively short half-lives of ^221^Fr  (T_1/2_ = 4.8 m) and ^213^Bi  (T_1/2_ = 45.6 m). On the other hand, the relatively long half-life of ^223^Ra  (T_1/2_ = 11.43 d) means most alpha decay counts of ^227^Ac progeny are measured after this time.

The e.o.c activities, $A_{e.o.c}(X_0)$, of the implanted ^227^Ac or ^225^Ac in each sample are related to the count rates, $R_{X_i}(t_{out})$, at the time the foil is removed from in front of the detector at time, $t_{out}$, by geometric and time-dependent nuclear decay feeding factors shown in eq. (4).

4 $$\begin{aligned} R_{X_i}(t_{out}) = A(t_{e.o.c}, Ac)\mathcal {F}_{B}(t_{in}, t_{out}, X_j)\epsilon _{\alpha }^{d\rightarrow d}(X_i) \epsilon _{X_i}^{f \rightarrow d}(X_0) \end{aligned}$$

$\epsilon _{X_i}^{f \rightarrow d}(X_0)$ is the probability for the *i*^th^ alpha-decay daughter, $X_i$ to recoil onto the detector following decay of its parent, $X_0$ (^227^Th or ^225^Ac), and any subsequent daughters (i.e. the probability of isotope *i* to occupy the ‘detector’ state in Fig. 1). $\epsilon _{\alpha }^{d\rightarrow d}(X_i) = 50$ % is the probability that the alpha particle from nuclide $X_i$ on the detector is detected in the detector. The Bateman decay and branching factor, $\mathcal {F}_{B}(t_{in}, t_{out},X_j)$, is the activity of isotope $X_j$ that has grown in during the implantation time, $t_{in}$ to $t_{out}$, due to decay of its parent(s) in the sample foil as a fraction of $A_{e.o.c}(X_0)$. $X_j$ is the parent of $X_i$ with the limiting half-life (e.g. $X_j$ = ^221^Fr for $X_i$ = ^217^At and $X_j$ = ^223^Ra for $X_i$ = ^219^Rn or ^215^Po). The geometric factors were calculated with SRIM^52^ simulations, as explained in section 2 in the supplementary material. The ^227^Ac activity was then calculated using two methods.

The first method uses the ratio of the alpha particle count rate on the detector of a given ^227^Ac progeny, $Y_{i}$, at the time the sample was removed, to that of the equivalent alpha-decay generation daughter of ^225^Ac, $X_{i}$. Furthermore, it exploits the very similar geometric factors for second and third generation decay daughters between the ^227^Ac and ^225^Ac decay chains. For example, for a sample-detector distance of 9 mm, the product of the geometric factors were 3.50% and 3.57% respectively for ^219^Rn and ^217^At and 2.74% and 2.76% respectively for ^215^Po and ^213^Po. This is due to the similar recoil energies in the two decay chains. Consequently, the geometric factors can be considered to be almost equal for the two decay chains. By ignoring this negligible difference in geometric factors, the ^227^Ac e.o.c activity can be calculated from eq. (5) for each sample, in terms of the ^225^Ac e.o.c. activities shown in table 3, the ratio of the aforementioned e.o.i. count rates, and the Bateman decay and branching ratio fractions.

5 $$\begin{aligned} A(t_{e.o.c.}, ^{227}Ac = \frac{R_{X_{227}}(t_{out})}{R_{X_{225}}(t_{out})}\frac{\mathcal {F}_{B}(t_{in}, t_{out}, X_{225})}{\mathcal {F}_{B}(t_{in}, t_{out}, X_{227})}A(t_{e.o.c.}^{227}Ac) \end{aligned}$$

The advantage of this method is that it is practically geometric efficiency independent due to the similarity of the recoil dynamics in the ^225^Ac and ^227^Ac decay chains. This greatly reduces systematic effects from the complex geometric efficiencies of alpha-decay chains. Furthermore, the ratio of ^227^Ac activity to ^225^Ac activity is directly determined without need for a-priori knowledge of the ^225^Ac activity.

The second method to calculate the ^227^Ac activity made direct use of eq. (4), with explicit calculation of the geometric efficiencies and the Bateman decay and branching ratio fractions.

### Results

Alpha-decay spectrometry on the alpha-decay recoil progeny of ^227^Ac and ^225^Ac was performed for each of the sample foils following the three stages of accumulation, implantation and measurement shown in Fig. 1. The accumulation time for sample A, B and C was 53.1 d, 32 d and 67.6 d respectively. The samples were mounted inside the chamber and recoil implantations were then performed at respective source detector distances of 7 mm, 13.5 mm and 9 mm for durations of 20.9 d, 38 d and 25 d respectively. Finally, the decays of alpha-decay recoil progeny were measured for durations of 10 d, 19 d and 14 d respectively. The relative implantation to measurement time durations were chosen to maximize overall counting statistics of alpha particles from alpha decay recoil progeny of ^227^Ac, while the absolute times dedicated to each experiment were determined by facility constraints. The source-detector distances were chosen on a case-by-case basis to achieve sufficient alpha decay energy resolution while maximizing counting statistics for ^225^Ac yet ensuring that the alpha particle count rate did not exceed approximately 1 % of the reciprocal detector dead-time.

**Fig. 2:**
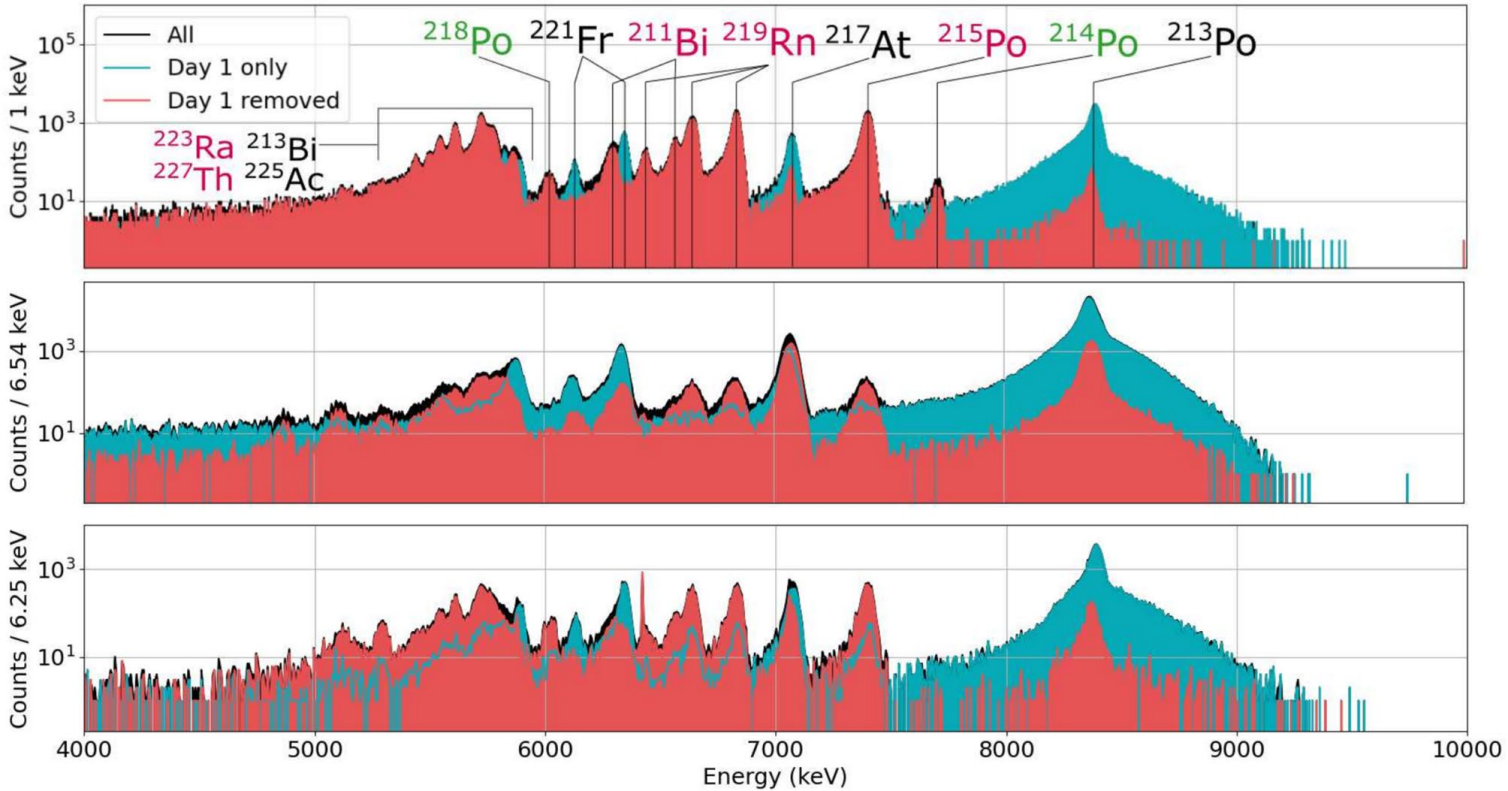
Measured alpha-decay recoil spectra over the experimental campaigns for samples A (above), B (middle) and C (below). After one day of measurement, the spectra are dominated by the ^221^Fr and daughter alpha decays (blue). In the remainder of the measurement time, few further counts of ^221^Fr and daughter peaks were recorded, while ^223^Ra and daughter alpha-decay peaks appeared (black). ^223^Ra and daughter alpha-decay peaks are identified in the spectra once the data of the first day are removed (red).

Decay data was collected for the full duration of the implantation and measurement stages of the experiment for each sample foil. The PIPS detector signal was pre-amplified and connected to a CAEN 6724c digitizer which output time-stamped signal strengths to a computer. Typical alpha-decay spectra of the full recoil measurements are shown in Fig. 2 for each sample. The grow-in of ^227^Ac daughters in the spectrum became visible after the first day of measurement, while the peak counts of the ^225^Ac daughters did not significantly increase. The figure shows how the recorded ^225^Ac daughter alpha-decay counts are greatly reduced by removing the data from the first day of the measurement, corresponding to 300 and 32 half-lives of ^221^Fr and ^213^Bi respectively, such that all ^225^Ac daughters implanted on the PIPS detector were expected to have decayed. Alpha decay counts due to decay of ^225^Ac progeny were nonetheless recorded. This remaining background is due to a combination of recoil self-sputtering and the ‘ping pong’ effect. In recoil self-sputtering, momentum transfer from alpha decay recoil daughters in the sample foil trigger collision cascades that can eject atoms from the sample, including some atoms of ^225^Ac. During the implantation phase, a small fraction of ^225^Ac atoms are recoil sputtered onto the detector surface where they contribute to alpha decay background during the measurement phase. In the ‘ping-pong’ effect, alpha particles from ^217^At and ^213^Po are measured from the distant sample due to consecutive recoil of a daughter nuclide from the sample, then back-ejection of its daughter from this surface.

The relative contributions of these two mechanisms to the background was evaluated by removing the sample entirely from the chamber after the measurement phase of several days, thus removing the background contribution due to the ping-pong effect. The order of one alpha count of ^221^Fr, ^217^At and ^213^Po progeny per million ^225^Ac decays due to recoil-sputtered ^225^Ac was measured, while the number of ^217^At and ^213^Po alpha counts due to the ping-pong effect was approximately 10 per million ^225^Ac decays. Therefore, the latter most strongly limited the sensitivity of the measurement of ^227^Ac progeny. Nonetheless, a factor 10,000 improvement is calculated in relative sensitivity of ^227^Ac counts to ^225^Ac counts using this method. A full sensitivity analysis is provided in the supplementary material.

Despite this background, ^227^Ac progeny alpha decay peaks were resolved in the recoil decay spectra of each sample. In sample A and C, the 7386.1 keV (100%) ^215^Po and 6819.1 keV (79.4%) ^219^Rn peaks were prominent, while in sample B the latter was less prominent due to worse detector resolution. In the recoil spectra of sample A, peaks at 6003 keV and 7687 keV were also identified, attributed to decay of ^214^Po and ^218^Po respectively, originating from ^226^Ra decay.

Time spectra with energy gates on these prominent identified alpha-decay peaks, were produced and fitted with an exponential decay to determine the end of implantation time (e.o.i.) count rates, $R_{X}(t_{out})$, shown in Fig. 8 in the supplementary material. The resulting decay constants of the fitted curves corresponded closely in all cases to their longest-lived parent nuclides indicated in the ‘detector’ location of Fig. 1 confirming their origin from feeding of recoiled progeny on the detector and not the ^225^Ac or ^227^Ac in the sample foil. The fitted decay constants and e.o.i. count rates of each analyzed nuclide in each sample are summarized in table 2.

**Table 2:** Fitted apparent half-lives and count rates at implantation end time in the alpha-decay spectrometry of recoil daughter measurements.

	Sample A		Sample B		Sample C	
**Nuclide**	Apparent T_1/2_	Count rate (s^-1^)	Apparent T_1/2_	Count rate (s^-1^)	Apparent T_1/2_	Count rate (s^-1^)
^217^At	4.79(7) m	17.4(4)	5.06(7) m	21.0(4)	4.95(14) m	6.0(2)
^213^Po	45.90(14) m	36.8(2)	45.70(10) m	79.1(3)	45.95(9) m	13.23(5)
^219^Rn	11.5(3) d	0.0885(10)	11.2(8) d	0.00234(11)	11.1(9) d	0.0046(2)
^215^Po	11.6(3) d	0.0966(10)	9.91(7) d	0.00264(13)	11.1(9) d	0.0054(2)
^218^Po	7.0(8) d	0.0029(2)				
^214^Po	3.3(3) d	0.0021(2)				

The apparent half-lives of ^217^At and ^213^Po correspond the half-lives of ^221^Fr  (T_1/2_ = 4.806(6) m) and ^213^Bi  (T_1/2_ = 45.62(6) m)^51^ respectively, generally with good agreement. The apparent half-lives of ^219^Rn and ^215^Po each correspond to the half-life of ^223^Ra(T_1/2_ = 11.43(3) d)^53^ The apparent half-life of ^214^Po in sample A corresponds to the half-life of ^222^Rn (T_1/2_ = 3.8232(8) d), while the apparent half-life of ^218^Po was poorly determined due to overlap of its analyzed 6003 keV peak with others in the alpha-decay energy spectrum.

The ^227^Ac activity was determined using eq. (5) with $Y_{i}$ = ^219^Rn and $X_{i}$ = ^217^At. The resulting ^227^Ac e.o.c. activities obtained are 3.90(13) Bq, 0.158(7) Bq and 0.24(4) Bq for samples A, B and C respectively. This method could not be used for subsequent generation Po nuclides as the only contribution to measured ^215^Po alpha counts is from implanted ^223^Ra whereas measured ^213^Po counts are due to feeding from implanted ^213^Bi, ^217^At and ^221^Fr on the detector. Therefore a simple exponential fit could not be performed to obtain an e.o.i. count rate of ^213^Po.

The ^227^Ac activity was additionally calculated using eq. (4). Geometric efficiency factors and the Bateman decay and branching factor for the 6819.1 keV ^223^Ra and 7386.1 keV ^215^Po peaks were calculated for each of the samples. The resulting ^227^Ac activities were 3.7(2) Bq, 0.15(1) Bq and 0.22(5) Bq for samples A, B and C, using the ^219^Rn peak and 4.1(2) Bq, 0.17(2) Bq and 0.27(5) Bq using the ^215^Po peak.

The $\alpha -srp$ approach was validated by performing direct alpha-decay spectrometry on samples A and C at times of 297 and 276 days after e.o.c. respectively, by which time ^227^Ac progeny peaks were prominent in the energy spectra. Sample B was not analyzed as it was used for another experiment. The ^227^Ac e.o.c. activity was obtained from analysis of alpha-decay energy peaks of ^215^Po, and ^219^Rn described in the supplementary material. The weighted mean ^227^Ac activity from the analysis for samples A and C were 3.70 (25) Bq and 0.248 (17) Bq respectively. These ^227^Ac e.o.c. activities are in very good agreement with those calculated with eq. (5), demonstrating the reliability of this method. The ^227^Ac activities calculated with eq. (4) are consistently slightly underestimated. This is likely due to a systematic overestimation of the absolute geometric efficiency terms. For this reason, only the ^227^Ac e.o.c. activities calculated using eq. (5) are used in further calculations.

## Activity measurements of ^225^Ac, ^225^Ra and contaminant nuclides

**Fig. 3:**
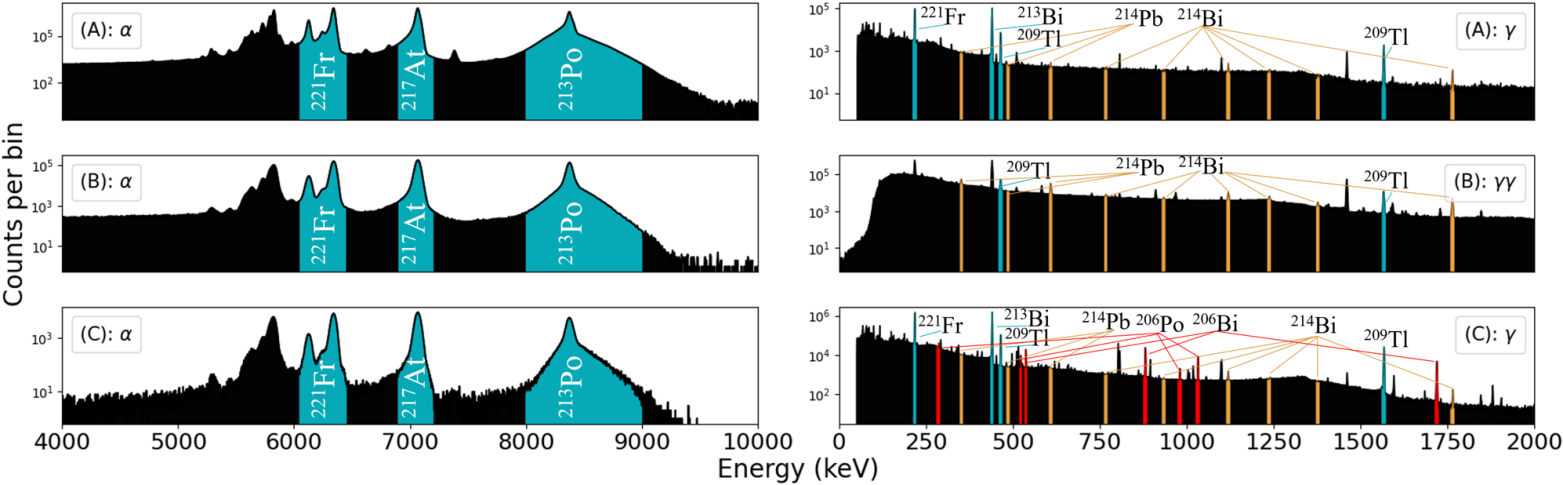
Nuclear-decay energy spectra for each of the samples. analyzed peaks in the ^225^Ac decay chain are highlighted in blue. $\alpha$-decay spectrometry: 6341 keV, 6126.3 keV and 6241.8 keV for ^221^Fr, 7066.9 keV for ^217^At and 8376 keV for ^213^Po. $\gamma$-decay spectrometry: 218 keV for ^221^Fr, 440.45 keV for ^213^Bi and 1567.08 keV and 465.14 keV for ^209^Tl. $\gamma \gamma$-coincidence spectrometry: 1567.08 keV and 465.14 keV for ^209^Tl. Several peaks of ^214^Pb and ^214^Bi daughters of ^226^Ra are due to background radiation (yellow). ^206^Po and ^206^Bi peaks for sample C were partially used to confirm presence of PoO (red). See supplementary material for details.

This section discusses the measurements of activities of ^225^Ac, ^225^Ra  (where applicable) and other contaminants identified in each sample. Samples A and C were measured with gamma-ray spectrometry and alpha decay spectrometry. Sample B was measured with alpha-decay spectrometry and gamma-gamma-coincidence spectrometry. Representative nuclear-decay energy spectra are given in Fig. 3. A detailed report on the methodologies for activity calculations presented in this section is provided in the supplementary material.

### Methods

Alpha-decay spectrometry was initially performed for each sample in the Alpha SETup (ASET) spectrometry chamber at the same time as performing the implantation for the $\alpha$-srp method. For each of the samples, the count rates of prominent ^221^Fr, ^217^At and ^213^Po peaks, shown in Fig. 3, were continuously measured and split into files of equal data size. The corresponding ^225^Ac activities for each file were calculated from the analyzed progeny peaks accounting for isotope-dependent geometric efficiency changes due to redistribution through alpha-decay recoil, as described in the supplementary material.

Samples A and C were again measured with alpha spectrometry in the ASET after long waiting times, as previously described in section 4.2, to detect long-lived radionuclidic impurities. The samples were measured for 4 days and 6 days respectively at a source-detector distance of 9 mm.

Gamma-decay spectrometry for samples A and C was performed in a lead castle with a coaxial Canberra high purity germanium (HPGe) detector. In each case the samples were placed at 100 mm from the detector casing in glass vials such that the center of the sample was a further 8 mm from the detector casing. Each campaign lasted at least one week, with measurement times of at least several hours per run.

The count rates of the 218 keV, 440 keV, 465 keV, and 1567 keV gamma rays following the decays of ^221^Fr, ^213^Bi, ^209^Tl, and ^209^Tl respectively were measured. In sample C, ^206^Po (T_1/2_ = 8.8 d) and its decay daughter, ^206^Bi (T_1/2_ = 6.2 d) were also identified through several peaks indicated in Fig. 3.

Gamma-gamma-coincidence spectrometry was performed for source B, to measure the ^225^Ac source activity independently of total detector efficiency. Two Canberra coaxial HPGe detectors were positioned at 16.5 and 18 cm respectively from the sample at an angle of 90$^\circ$  from one another. Both ^213^Bi and ^209^Tl decays are followed by gamma-decay cascades with coincident photons of sufficient intensity for these measurements. However, only the transitions following the decay of ^209^Tl have known multipolarities, so the coincidence data from ^213^Bi was not analyzed further. The measurement data was again split into files of equal size. The activity of ^209^Tl was calculated for each file at the mean time, *t*, using eq. (6).

6 $$\begin{aligned} A(t) = \frac{N_{i}[E_1](t) N_{ii}[E_2](t)}{N_{i,ii}[E_1, E_2](t)} \frac{I_{12, \gamma }}{I_{1, \gamma } \, I_{2, \gamma }} \frac{W(\pi /2)}{\left<W(\theta _{i,ii})\right>_{\theta }} \end{aligned}$$

Here $N_i[E_1](t)$ and $N_{ii}[E_2](t)$ are the count rates of photons labeled ‘1’ and ‘2’ in the gamma-decay cascade in detectors *i* and *ii* respectively. $N_{i,ii}[E_1,E_2]$ is the count rate of photons 1 and 2 measured in detectors *i* and *ii* respectively within a coincidence time window of 200 ns. *I* refers to the gamma-decay intensity per parent decay. $W(\pi /2)/\left<W(\theta _{i,ii})\right>_{\theta } = 0.95$ is the multipolarity correction at $\pi /2$ radians.

The obtained activity data of ^225^Ac decay progeny were in each case converted to in-sample ^225^Ac activity, accounting for decay intensities, branching ratios and secular equilibrium factors. The ^225^Ac e.o.c. activity in sample A was then calculated through fitting the activity data with an exponential decay. ^225^Ac activity data in samples B and C were in all cases fitted to account for possible ^225^Ra decay feeding using eq. (7).

7 $$\begin{aligned} A^{(B)}(t) = A_{e.o.c}^{(B)}e^{-\lambda _{B}t} + A_{e.o.c}^{(A)}\frac{\lambda _{B}}{\lambda _{B} - \lambda _{A}}\left( e^{-\lambda _{A}t} - e^{-\lambda _{B}t}\right) \end{aligned}$$

Here $\lambda _{A}$ and $\lambda _{B}$ are the ^225^Ra and ^225^Ac decay constants and $A_{e.o.c}^{(A)}$ and $A_{e.o.c}^{(B)}$ are the e.o.c activities of ^225^Ra and ^225^Ac respectively.

The ^206^Bi and ^206^Po activity data in sample C were fitted with the Bateman equation and an exponential decay, respectively, to yield the ^206^Po e.o.c. activity.

The obtained relative statistical error on the fitted e.o.c activity parameters were added in quadrature with the relative systematic errors on decay intensities and cumulative branching ratios and detector efficiency where applicable. A weighted mean of the e.o.c activities obtained from all analyzed decay lines for each spectrometry method was calculated. The dominating source of uncertainty in gamma-decay and alpha-decay spectrometry were detector efficiencies, while the ^225^Ac e.o.c. activity determined through gamma-gamma coincidence spectrometry was limited by statistics.

### Results

The fitted e.o.c. activity of ^225^Ac for all samples, and ^225^Ra in samples B and C are shown in table 3 for each spectrometry method. The weighted mean value from different methods is shown in bold. The fitted e.o.c. activity of ^225^Ra was determined to be negligible in sample C. The results are presented alongside the ^227^Ac e.o.c. activities calculated using eq. (5) for all samples, and the ^226^Ra e.o.c. activity in sample A calculated using eq. (4).

The ^206^Po e.o.c. activity of 980(40) Bq deduced from the weighted mean of analyzed ^206^Bi and ^206^Po gamma lines is shown for sample C.

In addition, e.o.c. activities of ^226,228^Ac and ^208^Po in sample C were deduced from the analyzed alpha decay data after long waiting time. The identified ^208^Po alpha decay peak has no candidate parent that could be implanted as a monoatomic ion beam in the mass range $222<A<228$. Its presence was thus attributed to implantation of ^208^PoO^+^.

^210^Po and ^214^Po peaks were identified, with calculated activities consistent with each other assuming decay of implanted ^226^Ac^+^. Details of this calculation are included in the supplementary material. Production through decay feeding of implanted ^226^Ra was ruled out, as negligible ^225^Ra was detected in the sample, and all radiogenic Ra isotopes in the target material were outgassed prior to collection. Finally, the ^212^Po alpha decay peak was attributed to the decay of implanted ^228^Ac^+^. ^228^Ra implantation was ruled out based on the same arguments as before. As it has been deduced that ^208^Po is implanted as an oxide sideband at $A/q = 224$, it was also expected that ions of the longest-lived Po isotope, ^209^Po, were implanted at mass 225 with a lower suppression factor. However, its alpha-decay peak was not observed. An upper limit was estimated using the Currie method to be 2.32(7) mBq, as shown in table 3.

**Table 3:** E.o.c activity of identified nuclides in the three collected samples. *Values are mean activities from all spectroscopy methods for ^225^Ac  ^225^Ra and ^226^Ra, and activity from eq. (5) for ^227^Ac. $^{\dagger }$Value consistent with 0 within error. $^{\ddagger }$Values correspond to the Minimal Detectable Activity (MDA) calculated with the Currie method.

Sample	Nuclide	E.o.c activity (kBq)					
		$\alpha$ / $\alpha _{long}$	$\gamma$ / $\gamma \gamma$	$\alpha$-srp eq. (5)	$\alpha$-srp eq. (4)	Final^*^	$f_{sep}$
** A**	^225^Ac	57.8(23)	51.3(14)			**52.9(12)**	
	^227^Ac			3.90(13)$\times 10^{-3}$	3.19(9)$\times 10^{-3}$	**3.90(13)**$\times 10^{-3}$	$1.26(8) \times 10^2$
	^226^Ra				6.2(4)$\times 10^{-5}$	**6.2(4)**$\times 10^{-5}$	
**B**	^225^Ac	79.0(10)	103(47)			**79.0(10)**	
	^225^Ra	11.3(12)	30(20)			**11.3(12)**	
	^227^Ac			1.58(7)$\times 10^{-4}$	1.14(6)$\times 10^{-4}$	**1.58(7)**$\times 10^{-4}$	$9.9(7) \times 10^2$
** C **	^225^Ac	97.1(38)	88.6(25)			**91.2(21)**	
	^225^Ra					**0**$^{\dagger }$	-
	^226^Ac	2.2(3)				**2.2(3)**	$2.2(3) \times 10^{2}$
	^226^Ra				0$^{\dagger }$	**0**$^{\dagger }$	-
	^227^Ac			2.4(4)$\times 10^{-4}$	1.96(13)$\times 10^{-4}$	**2.4(4)**$\times 10^{-4}$	$6.0(9) \times 10^2$
	^228^Ac	$3.2(2) \times 10^{-2}$				**3.2(2) **$\times 10^{-2}$	$2.6(2) \times 10^4$
	^206^Po		0.98(4)			**0.98(4)**	$2.4(2) \times 10^2$
	^208^Po	1.25(5) $\times 10^{-4}$				**1.25(5) **$\times 10^{-4}$	$1.45(8) \times 10^4$
	^209^Po$^{\ddagger }$	2.32(7) $\times 10^{-6}$				**2.32(7) **$\times 10^{-6}$	$2.17(11)\times 10^4$
	^210^Po	$0^{\dagger }$				**0**$^{\dagger }$	-

## Discussion: Purity and separation enhancement factors

**Fig. 4:**
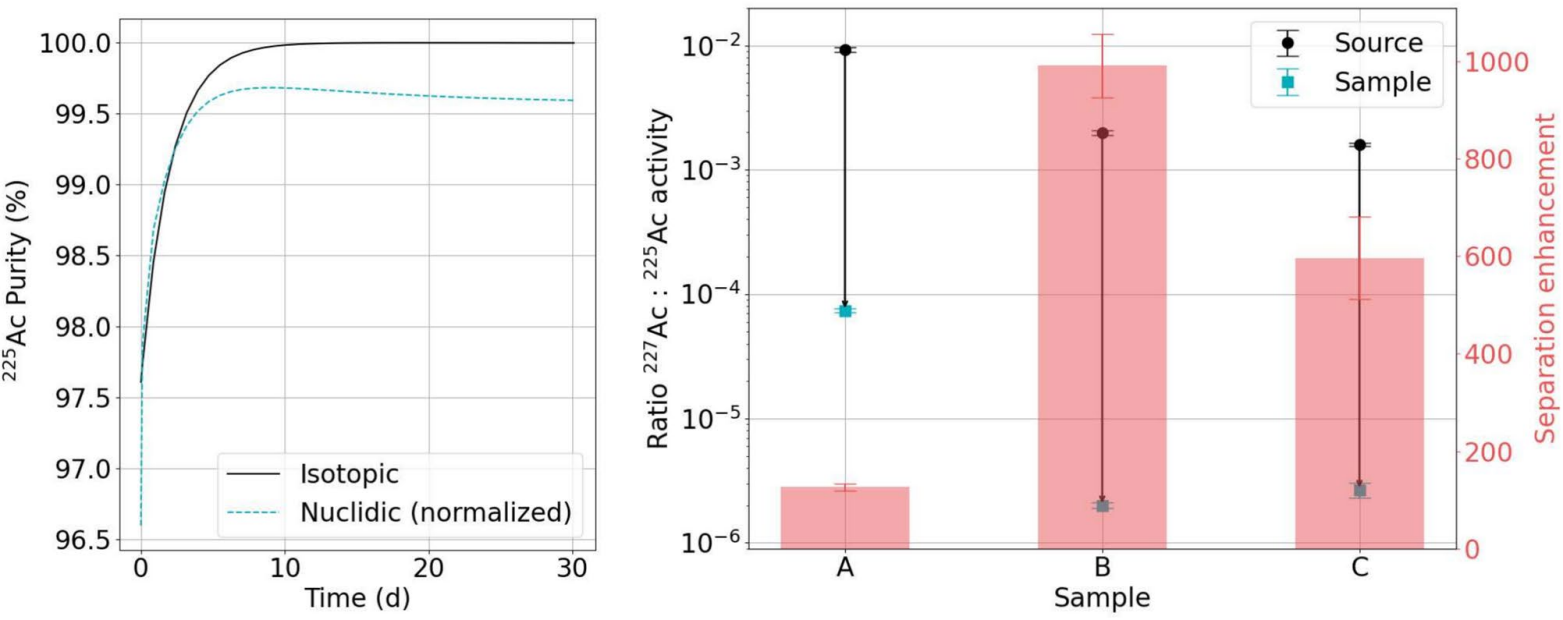
Purity of ^225^Ac samples.

The radionuclidic purity, $p_{rn} = 96.6(3) \%$ and radioisotopic purity, $p_{ri} = 97.7(3)\%$ of ^225^Ac at e.o.c. were calculated for sample C based on the activities of implanted contaminants shown in table 3. The latter is dominated by the ^226^Ac content that decays with a half-life of 29.37(12) h. After 2 days, $p_{ri} > 99\%$, and after 1 week, $p_{ri} > 99.9 \%$. The radioisotopic purity of ^225^Ac and the radionuclidic purity of ^225^Ac and its decay progeny as a function of time are shown in Fig. 4a.

The separation enhancement factors of ^225^Ac with respect to the identified nuclides in the decay spectrometry campaigns of this work are shown in the final column of table 3. The separation enhancement factor with respect to ^226^Ac is in line with expectations, outlined in section 3.2, while the separation factor with respect to ^227^Ac is lower than expected. Similarly, the separation enhancement factor of $2.6(2) \times 10^{4}$ with respect to ^228^Ac is lower than would be expected according to the arguments in section 3.2, though not problematic for medical applications.

The low separation enhancement factor of ^225^Ac with respect to ^206^Po was not initially expected, though can be explained through parasitic implantation due to the sample foil positioning. This is the mechanism by which beams of *A*/*q* different to those selected by the mass separator, yet still transmitted through the separator slit, are implanted into the sample foils adjacent to the targeted sample foil.

Here, sample foil C was situated in the middle position of the sample holder (pos 2) (shown for example in Fig. 3 in ref.^36^). For 96 % of the collection time, the beam of $A/q = 225$ was implanted onto a targeted foil that was positioned to the higher mass side (pos 3) of sample C. During this implantation, the foil of sample C may have been exposed to transmitted ions of $A/q < 225$. The transmission factor for such parasitically implanted ions would be maximum for a few mass-to-charge units lower than $A/q = 225$, explaining the enhanced amount of ^206^Po in sample C. The ^208^Po oxide sideband, on the other hand, is more likely implanted during the time when foil C is targeted by the $A/q = 225$ beam. This work shows that Po isotopes are not implanted with high enough rate to be problematic for medical applications of the ^225^Ac sample. In any case, a radiochemical separation step is required to recover the ^225^Ac from the Al foil material. The mechanism of formation of PoO^+^ beams warrants further study.

Using the e.o.c activity values calculated for ^225^Ac and ^227^Ac, the separation enhancement factors of ^225^Ac over ^227^Ac were determined for samples A, B and C to be 126(8), 990(70) and 600(85), while the e.o.c activity ratios of ^227^Ac to ^225^Ac were calculated as 7.4(3) $\times 10^{-5}$, 2.00(10) $\times 10^{-6}$ and 2.7(4) $\times 10^{-6}$ respectively. The results are presented in Fig. 4b.

The separation enhancement factor of ^225^Ac over ^227^Ac varies by an order of magnitude between the samples. A plausible explanation to account for some of this difference is again due to parasitic implantation as a consequence of sample foil position in the collection chamber. When the mass separator is set to collect a beam of $A/q = 225$, the calculated beam centroid position of ^227^Ac at the focal plane is 10.4 mm to the high mass side of the targeted foil center. The center of the adjacent foil is a further 14.6 mm from the centroid of the ^227^Ac beam. Therefore, depending on the transmitted ^227^Ac beam shape, some fraction of ions are transmitted to the adjacent sample foil on the high-mass side in addition to the targeted sample foil. In the cases where the majority of the beam is implanted on an adjacent foil, this parasitic implantation can bias the subsequently measured separation enhancement factors.

The foil of sample B was placed in pos. 1 at the lowest mass side of the sample holder, so no parasitic implantation of higher mass beams was possible. Sample foil C, on the other hand, was placed in pos. 2. During this collection, implantations of $A/q = 225$ were performed on the sample foil in pos. 1, to the ‘low mass’ side of the sample C foil, at 31.5 and 47.5 hours into the collection, each for a duration of 30 minutes. This leads to a potential extra contribution of ^227^Ac implanted into the foil of sample C through parasitic implantation, consistent with a lower value of $f_{sep}$. For sample A, no implantation was performed on the adjacent foil on pos. 2 meaning parasitic implantation due to the above described mechanism was unlikely. However, for several hours during this collection, the beam of $A/q = 227$ was implanted on the foil placed in pos. 1. Although this beam was never directly implanted on the foil of sample A, it cannot be excluded that some ions of transmitted ^227^Ac were scattered onto it.

In light of these considerations, it is argued that sample B gives the most representative value of $f_{sep}$ of ^227^Ac over ^225^Ac due to the absence of parasitic implantation. This value is therefore recommended to apply to MEDICIS collections in the future, when the beam is not implanted for a large fraction of the collection time on neighboring foils.

Aside from the above discussed differences between the collections, there are several additional systematic effects that can lead to potentially large variability in the separation enhancement factors, $f_{sep}$. For example, small deviations on the order of 3 % from the optimum value of Einzel lens voltage in the MEDICIS separator beamline can lead to a decrease of the mrp by up to a factor of two^36^, that can significantly impact the value of $f_{sep}$. Moreover, the mrp can also be influenced through different beam emittences. This may apply to the collection of sample A, that was performed at 50 kV extraction voltage instead of 60 kV, where the lower energy of the extracted ions can worsen the beam emittance. In addition, the precise positioning of the acceptance slits is also a variable that influences $f_{sep}$, that is optimized during each collection, but may not always be reproduced between collections. Another mechanism could be co-implantation of a parent nuclide, such as feeding from implanted ^225^Ra that increases measured ^225^Ac e.o.c. activity. Here it can be excluded that the higher $f_{sep}$ for sample B is due to feeding from ^225^Ra present in the sample. Even if all of the measured 11.3(12) kBq was implanted at the start of the collection, only 3 kBq ^225^Ac would be generated in the collection time, accounting for 4 % of the separation enhancement factor.

Although the separation of ^225^Ac is mostly achieved by the separator magnet, some degree of isotope selectivity is also possible thanks to the resonance laser ionization scheme that has an isotope shift of 8 GHz between the 6d7s^2^ ^2^D_3/2_ $\rightarrow$ 6d7s7p ^4^P_3/2_^o^ first step transitions of ^227^Ac and ^225^Ac^41,54^. The lasers used in this work were set up such that the first step transition was at 22801.2906 cm^-1^, whereas the ^225^Ac centroid calculated from refs^41^ and^54^ was at 22801.5714 cm^-1^. The laser scheme used thus corresponds more closely to the resonant peak of ^227^Ac over ^225^Ac meaning that in principle, the enhancement could be marginally improved further with more precise frequency selection. In practice, there are often drifts in frequency on the order of tenths of inverse centimeters during operation. These drifts can further impact the separation enhancement factor of ^225^Ac over ^227^Ac on the order of several percent, that may further contribute to the discrepancies of $f_{sep}$ between samples. It is possible to control the frequency more precisely by incorporation of a feedback loop from the frequency readout to the frequency selective grating of the Ti:Sa laser cavity, which could help with ensuring reliable enhancement factors in the future.

The ratio of e.o.c. activity of ^227^Ac to ^225^Ac for the samples collected from irradiated targets (B and C) are in each case around three orders of magnitude below the typical ^227^Ac in ^225^Ac samples produced by radiochemical separation. Using the separation enhancement factor of sample B, it can be estimated that a typical patient dose of 10 MBq would contain approximately 20 Bq of ^227^Ac if the implanted beam was purely ^225^Ac. In practice, both ^225^Ra and ^225^Ac are implanted during a full collection, which means that the ratio of ^227^Ac to ^225^Ac in a sample collected by this method for production purposes would be even lower than calculated here, due to decay feeding from ^225^Ra, that increases the fraction of ^225^Ac to ^227^Ac in the foil. Similarly, the radioisotopic purity of ^225^Ac in such samples would be higher than that calculated for a pure ^225^Ac sample in this work.

## Conclusion

An extensive nuclear decay spectrometry campaign has been performed on samples of resonance-laser-ionized and mass-separated ^225^Ac produced from high-energy proton spallation of thorium-based targets at CERN-MEDICIS to quantify the radionuclidic and radioisotopic purity. Long-lived decay progeny of implanted contaminants have been identified in one of the samples. The activities of ^206, 208, 210, 212, 214^Po were measured and attributed to implanted ^206,208^PoO^+^, and ^226,228^Ac^+^.

A decay spectrometry method to measure alpha-decay recoil progeny of ^225^Ac and ^227^Ac was developed to evaluate the trace ^227^Ac activity with a 10,000-fold sensitivity improvement over standard decay spectrometry. The ^227^Ac activity in the two mass-separated samples collected from irradiated targets were determined to be 0.000200(10) % and 0.00027(4) % that of the ^225^Ac at end of collection respectively. The ^227^Ac to ^225^Ac activity ratio in MEDICIS collections of ^225^Ac exploiting co-implantation of ^225^Ra^+^ and ^225^Ac^+^ is expected to be significantly lower, depending on the ratio of implanted ^225^Ra^+^ to ^225^Ac^+^ ions. The calculated separation enhancement factor of ^225^Ac over ^227^Ac that corresponds to representative ^225^Ac^+^ collections performed at MEDICIS was 990(70). Samples produced with this method thus have a thousandfold reduction of ^227^Ac content compared to samples produced by direct chemical separation that offer no enhancement of directly-produced ^225^Ac over ^227^Ac following in-target production. The resulting radioisotopic purity of ^225^Ac at end of collection was $p_{ri} = 97.7(3)$ %, that was dominated by ^226^Ac, identified through alpha decays of ^210,214^Po progeny. With a cooling time of 2 days after end of collection, $p_{ri} > 99$ % is achieved. For clinical batch activities of 10 MBq ^225^Ac produced at CERN-MEDICIS through co-implantation of ^225^Ra^+^ and ^225^Ac^+^ the ^227^Ac activity fraction of 0.000200(10) % is an upper limit, and the radioisotopic purity of $p_{ri} > 99$ % after 2 days is a lower limit. While this study has demonstrated the high purity of mass-separated ^225^Ac samples, radio-labeling studies are required to evaluate the suitability of ^225^Ac-based pharmaceuticals based on mass-separated samples produced at CERN-MEDICIS for medical application.

## Supplementary Information

Supplementary Information.

## Data Availability

The data generated and analyzed for the current study are available from the corresponding authors on reasonable request.
