# Supplementary material for: Quantification of trace 227Ac and other radionuclidic impurities in mass-separated 225Ac samples produced at CERN-MEDICIS
Source: Sci Rep. 2025 Jul 2;15:23563. doi: 10.1038/s41598-025-02277-4 (PMC12222749; doi:10.1038/s41598-025-02277-4)
Supplement: Supplementary file 1 — Supplementary Information. [file 41598_2025_2277_MOESM1_ESM.pdf]

Supplementary material: Quantification of trace ^{227}Ac and other radionuclidic impurities in mass-separated ^{225}Ac samples produced at CERN-MEDICIS

J. D. Johnson[1 ✉], C. Bernerd[1,3], F. Bruchertseifer[2], T. E. Cocolios[1], M. Deseyn[1], C. Duchemin[1,3], M. Heines[1], M. Keppens[1], L. Lambert[3], N. Meurrens[1], R. E. Rossel[3], T. Stora[3], and V. Van den Bergh[1]

[1] KU Leuven, IKS, Leuven, Belgium
[2] JRC, Karlsruhe, Germany
[3] CERN, Geneva, Switzerland
✉ jake.johnson@kuleuven.be

ABSTRACT

This supplementary material serves to provide additional information on collections and decay spectrometry of mass separated samples produced in this work. The technical details including mass scans and beam composition evaluation of the collections of each sample are presented in section 1. In section 2, the SRIM simulations that were performed to model the geometric efficiencies for alpha particle detection of nuclides in the ^{227}Ac and ^{225}Ac alpha-decay chains are discussed. Fitted histograms of alpha particle count rates from ^{221}Fr and ^{223}Ra decay chains used for determining the count rates at end of implantation in the alpha-decay spectrometry of recoil progeny measurements are presented in section 3. Alpha spectrometry data for ^{225}Ac and ^{225}Ra activity measurement are reported in section 4.1, while the data taken after long waiting times for ^{227}Ac and other long-lived contaminant identification are given in section 4.2. The γ spectrometry data for ^{225}Ac and ^{225}Ra activity measurement is provided in section 4.3, where identification and quantification of other contaminants ^{226}Ra and ^{206}Po are also discussed. Extra details on $\gamma\gamma$ coincidence spectrometry data are given in section 4.4. Finally, the sensitivity analysis of the alpha-decay spectrometry of recoil progeny relative to direct alpha-decay spectrometry for ^{227}Ac detection is provided in section 5.

1 Technical details of sample collections

In section 3 in the main text, the experimental conditions under which the samples were collected were briefly discussed. Here, further technical details are given for each collection in order to provide information on the ion beam composition in the mass region of interest, as well as to illustrate mass-tailing structures that are visible from intense beams in the mass scans.

1.1 Sample A collection

Source A was loaded into the target unit after the target unit conditioning period described in the main text. Concurrently, three Ti:Sa lasers were set up and optimized according to the scheme described in the main text. After the target was placed on the front end, vacuum was established. The ion source was brought to 2040 °C, while the target was brought to 1600 °C for the initial period of outgassing. It was observed that the pressure inside the target vessel did not quickly decrease to below the usual 10^{-5} mbar limit needed to start operation. Even after it did, sudden increases in pressure above 10^{-5} mbar regularly caused the high voltage to discharge, meaning that the collection had to be performed with an extraction voltage of 50 kV instead of the usual 60 kV.

After the outgassing period, the target temperature was gradually increased, with several mass scans performed during this time, an example of which is shown in fig. 1. Several peaks due to ion beams formed from the ThO_2 target material were visible. Ion beams with mass to charge ratios, A/q = 232, 248 and 250 were observed corresponding to $^{232}Th^+$, $^{232}Th^{16}O^+$ and $^{232}Th^{18}O^+$ respectively. A beam at A/q = 217 corresponding to the molecular break-up peak of $^{232}Th^{16}O^+ \rightarrow {}^{232}Th^+ + {}^{16}O$ was also observed. Finally, a peak at A/q = 267 was visible which is hypothesized to be $^{232}Th^{35}Cl^+$. The presence of Cl can be explained due to the injection of Cl_2 as an oxidizing (volatilization) agent in the previous collection performed at MEDICIS. This could also explain the pressure spikes observed at the beginning of the target heating, as residual Cl_2 gas could have acted to rapidly volatilize impurities at low temperatures. Although the mass scan did not cover the full lanthanide region (M = 130 - 175), the presence of ions with beam currents of several tens of nA are expected in this region. Scattered ions from these beams lead to

the tailing background visible from masses 180 - 240. The background due to these beams on mass 225 is of the order 0.5 pA.

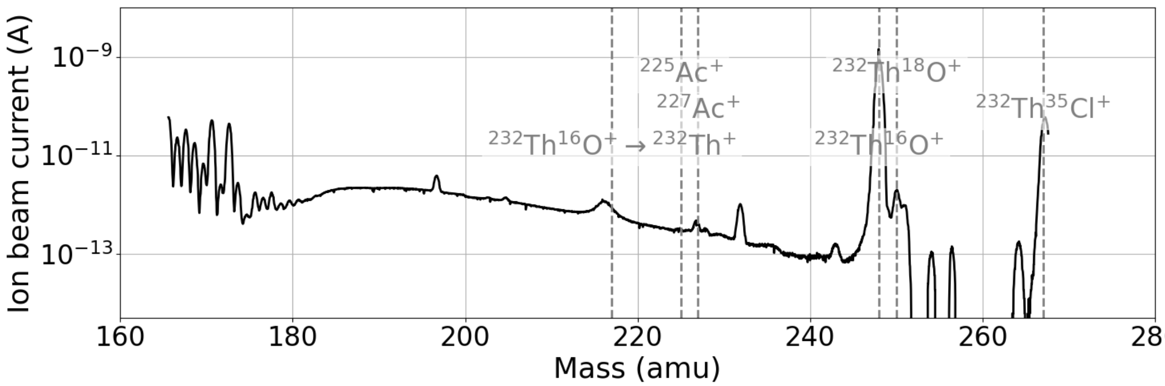

Figure 1. Mass scan performed during collection of sample A in the region 165 < A < 270. 42.5 hours since start of collection at target temperature of 2180°C. The total beam is dominated by $^{232}Th^{16}O^+$ in this region, and $^{225}Ac^+$ and $^{227}Ac^+$ are not visible due to the mass tailing of more intense beams colliding with residual gas molecules in the beamline.

A signal due to Ac^+ in the ion beam was first observed in a laser ON/OFF test of the beam incident on the separated beam Faraday cup, with the separator magnetic field set to A/q = 227. The signal was observed at a target heating current of 720 A corresponding to a temperature of 2160 °C. Once this was confirmed, the collection of $^{225}Ac^+$, began, 39.5 hours after the source was loaded onto the front end. An overnight pressure spike during the collection caused the extraction voltage to be discharged, meaning that for 6 hours, no beam was implanted. The target was heated up to a current of 870 A, which was the maximum used during this collection. The corresponding temperature of 2430 °C, according to the temperature calibration, is much higher than typically applied to this design of target. This was perhaps detrimental, as the target container failed towards the end of the collection with no heating current able to be applied. The total time during which ^{225}Ac was collected on sample A was 59.5 hours, out of the 111 hours allotted to the collection.

1.2 Sample B collection

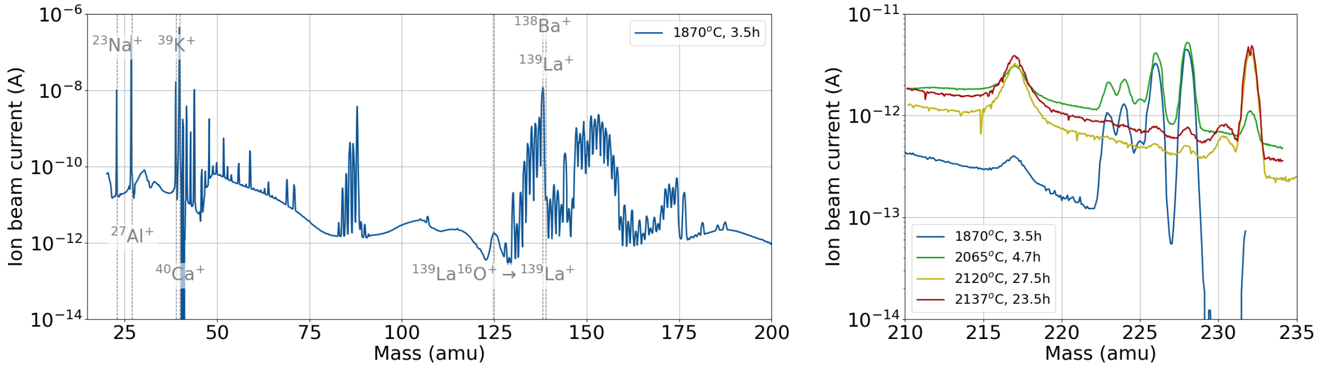

(a) Full range scan performed at a target temperature of 1870 °C

(b) Ra^+ and Th^+ beams in the A/q = 225 region.

Figure 2. Mass scans during the collection of sample B.

Source B consisting of the irradiated ThO_2 target was loaded onto the front end. The lasers were set up to the same scheme as for collection of sample A. The powers of the steps were 330 mW, 800 mW and 830 mW for steps 1, 2a and 2b respectively. During the outgassing period, while the target was at 1850 °C the ion beam composition was evaluated with a full range mass scan as seen in fig. 2. The beam was dominated by a range of impurities, that also gave rise to tailing structures due to interactions of intense beams with residual gas molecules in the beamline. Notably, very intense Na^+, Al^+, K^+ and Ca^+ beams due to target and target unit material impurities were observed in the low mass region, dominating the total beam. The presence of stable La^+ is likely based on its atomic ion peak at A/q = 139 and the broad LaO^+ molecular break-up peak at A/q = 124.7. Several intense beams of surface-ionized Ba^+ (A/q = 130 and $134 \leq A/q \leq 138$) were observed with a plethora of lanthanide

(LaX) beams ($139 \leq A/q \leq 175$). These most intense beams exhibit tailing that extends several dozens of masses and leads to a high (mostly > pA) background over almost all mass regions, and a background of the order of 1 pA at A/q = 225 for target temperatures above 2060 °C. At higher temperatures, mass scans performed in the mass range of interest, shown in of fig. 2b demonstrate that heating increased the contribution of the tailing from intense beams at distant masses on the mass of interest, but also that these beams were slowly diminishing with time.
Due to this tailing that gave rise to a background current on A/q = 225, it was difficult to confirm the presence of $^{225}Ac^{+}$ until the target was heated to 2260 °C, where the laser ionized $^{225}Ac^{+}$ current was higher than the background. More details of this collection are reported in ref. 1. Here, it suffices to summarise that $^{225}Ac^{+}$ was identified both through a resonant laser ON/OFF effect, as well as through the analysis of the 440 keV gamma ray of ^{213}Bi on the sample foil during the collection that was recorded in real time with a kromek GR-01 gamma ray spectrometer. In addition to $^{225}Ac^{+}$, $^{225}Ra^{+}$ was also collected. Online quantification of collected $^{225}Ra^{+}$ during the collection was challenging due to lack of direct detectable gamma radiation.

1.3 Source C conditioning

Figure 3 presents a representative summary of the 15 mass scans performed during the 6-day conditioning period before the target was irradiated. With heating and over time, the beams at A/q = 224, 226, and 228 diminish to currents barely above the tailing background. These are attributed to $^{224, 226, 228}Ra^{+}$, which are present in the decay chains of ^{232}Th and ^{238}U. On the other hand, the beams of $^{232}Th^{+}$ and $^{232}ThO^{+}$, as well as $^{238}U^{+}$ become more intense with heating and do not diminish over time. Similarly, the plethora of LaX beams produced from the ThC_x target material did not diminish over time - meaning that the mass tailing from these also does not significantly decrease. Finally, the intense beams visible in the low-mass region at low temperatures decrease significantly with heating and time, lowering their contribution to the total beam. At the end of the conditioning period the total beam (mostly LaX) was 27 nA. The ion current background due to mass tailing at A/q = 225 was 0.25 pA, with the target heating current of 730 A corresponding to 2138 °C.

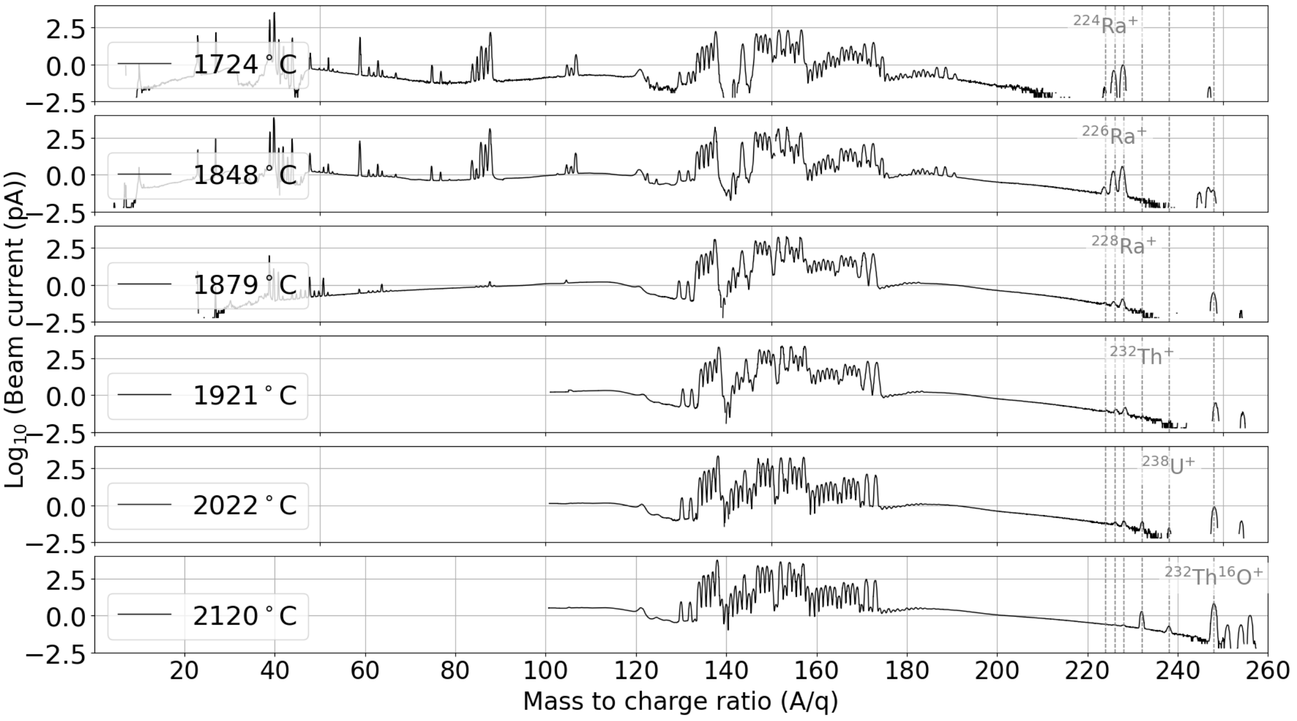

Figure 3. Mass scans taken over the 6 days of ThC_x conditioning at different target container temperatures.

section 1.3 shows the ion current background at A/q = 225 during the conditioning of the target. A peak in the background is seen at a heating current of 500A, corresponding to when neighbouring mass Ra atoms were released. The background then remains low for the rest of the conditioning period.

1.4 Sample C collection

The ThC_x target was put onto the MEDICIS separator front end following the irradiation described in section 2.2. The same laser scheme was used as for sources A and B, with powers of 350 mW, 750 mW and 960 mW corresponding to steps 1, 2a and

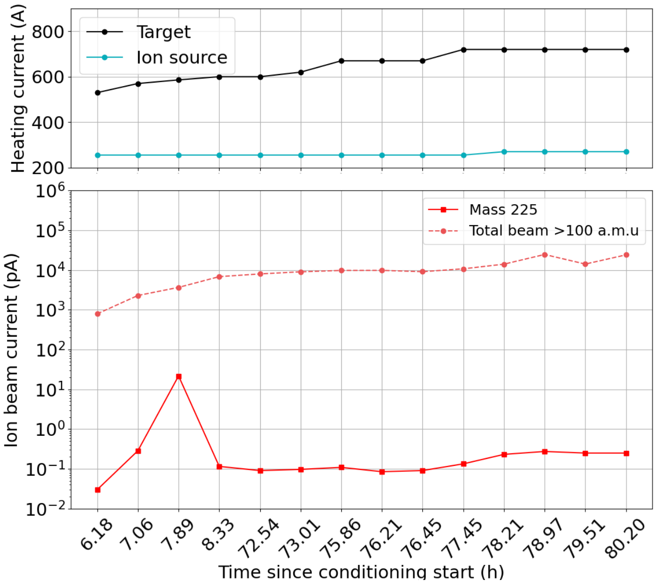

Figure 4. Beam currents due to all ions with A/q > 100 recorded from each mass scan during conditioning of the ThC_x target for source C. The peak in the A/q = 225 beam after 7.89 h corresponds to outgassing of neighboring mass Ra isotopes present in the target material, while the background at later times is due to tailing from LaX beams.

2b respectively. The ion source was heated with a current of 270 A to a temperature of 2110 °C. At the start of the collection, Ra^+ was quickly identified thanks to a mass scan with relative peak intensities on mass to charge ratios 220 < A/q < 230 that matched very closely with the in-target population ratio of the Ra isotopes, as shown in fig. 5. The target was gradually heated while $^{225}Ra^+$ was collected, with slits positioned at 9.5 mm and 1.5 mm to limit tailing contamination of other mass ion beams.

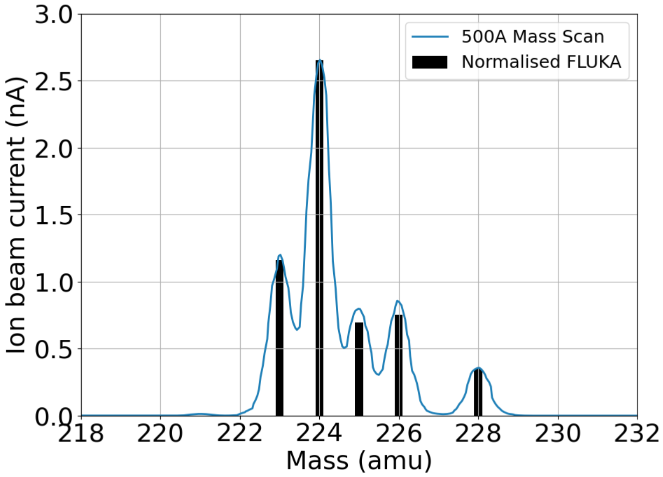

Figure 5. Mass scan in the mass 225 region at target temperature of 1650 °C 4.5 hours after collection start time for sample C. The FLUKA relative in-target population predictions correspond to a target irradiation time of 19 hours with 6 hours cooling time, and allowed identification of Ra^+ beam production

Several mass scans were performed during the collection, shown in fig. 6. At temperatures of 1400 - 1650 °C, the characteristic Ra^+ ion beam pattern of fig. 5 was observed. At 1980 °C ^{225}Ra beams are still visible, though with a slightly lower intensity. Furthermore, a second set of peaks that correspond to the ^{225}Ra in-target production yield ratios are seen, transposed 19 A/q units to the higher mass side. This is hypothesized to be $Ra^{19}F^+$ beams, though the origin of the ^{19}F is not clear. In the later mass scans taken at 2400 °C, the the Ra has been depleted and Ac^+ is visible at A/q = 227. Beams of A/q = 232, A/q = 238, A/q = 244 and A/q = 256 are visible, attributed to $^{232}Th^+$, $^{238}U^+$, $^{232}Th^{12}C^+$ and $^{232}Th^{12}C_2{}^+$ respectively.
At a target heating current of 800 A, corresponding to a temperature of 2260 °C, Ac^+ was first identified with a laser ON/OFF

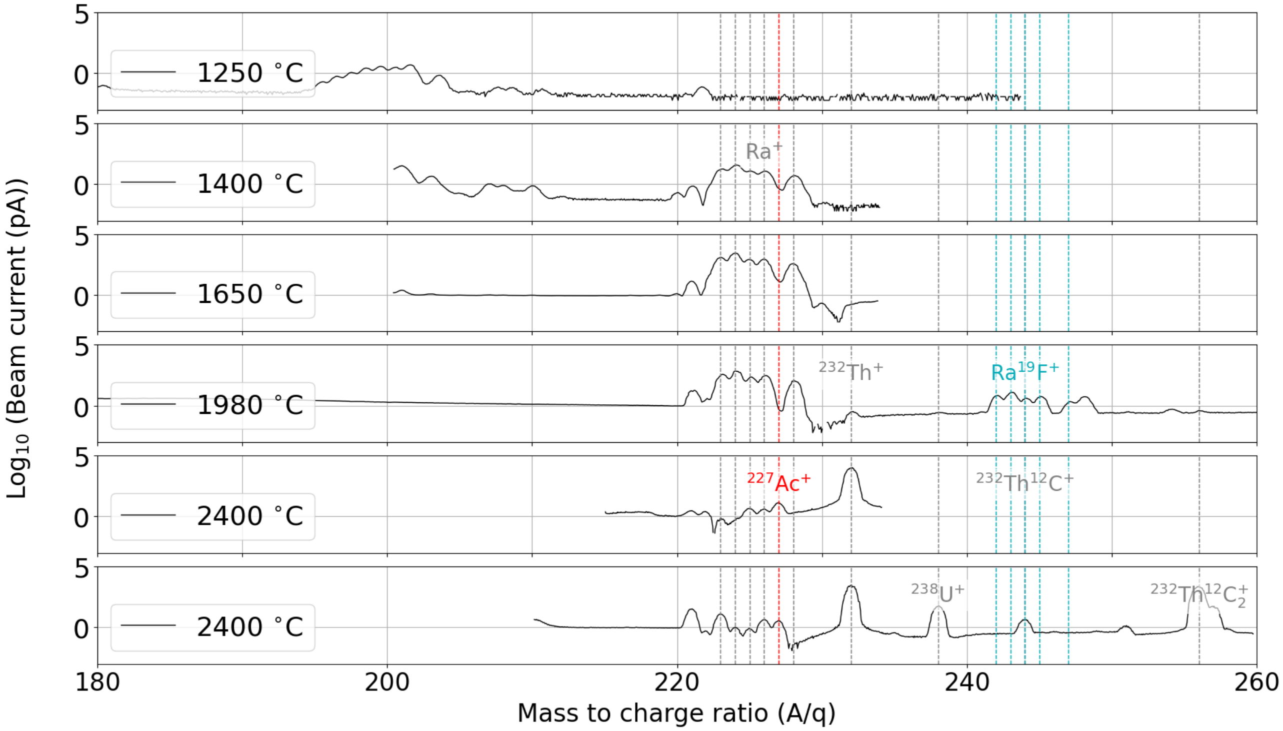

Figure 6. Mass scans performed during collection from source C. Intense Ra^{+} beams are visible at the lower temperatures of the collection (1400 - 1650 °C), that are then depleted. The beams in the $242 \leq A/q \leq 247$ range are tentatively assigned to $Ra^{19}F^{+}$. No long-range mass scan was performed at lower temperature to evaluate the presence of these beams. At target temperature of 2400 °C, Ac^{+} is visible at A/q = 227.

signal. The total ion beam current at this point was 100 nA. Once optimized, the laser enhancement was measured every ten minutes by blocking the lasers for 5 seconds with an automated shutter system. The laser enhancement remained close to a factor 2.4 for the remainder of the collection. The collection proceeded onto three collection foils, with 2x half-hour implantations on two foils to be sent to CHUV and KU Leuven for characterisation of ^{227}Ac contamination at 31 hours and 47 hours after the collection began. At these points, the ^{225}Ra had been depleted as descibed in the main text and the ion beam consisted primarily of $^{225}Ac^{+}$. For a short period during the collection the Einzel lens and deflectors were off which reduced the total collected $^{225}Ac^{+}$, but not on the CHUV sample nor sample C that was sent to KU Leuven. The collection ended after 56.3 hours.

2 SRIM simulations for ^{225}Ac and ^{227}Ac alpha decay progeny distributions

This section details the SRIM simulations that were performed to determine the relative geometric efficiencies of alpha decay progeny in the ^{225}Ac and ^{227}Ac chains.
Firstly, the depth distribution was simulated for both ^{225}Ac and ^{227}Ac in Al at 50 keV and 60 keV beam energies corresponding to collections A, then B and C respectively. For sources A and C, 100000 particles of ^{225}Ac and ^{227}Ac were respectively simulated, while for source B, 50,000 and 40,000 particles of ^{225}Ac and ^{227}Ac were simulated respectively. Once the initial depth distribution was calculated, the layered SRIM simulations were performed, using the mean recoil energies shown in table 1.

Generation	Decay ^{225}Ac chain	Recoil E (keV)	Decay ^{227}Ac chain	Recoil E (keV)
1	$^{225}Ac \rightarrow {}^{221}Fr$	105(3)	$^{227}Th \rightarrow {}^{223}Ra$	105.8(17)
2	$^{221}Fr \rightarrow {}^{217}At$	117.1(9)	$^{223}Ra \rightarrow {}^{219}Rn$	102.8(15)
3	$^{217}At \rightarrow {}^{213}Bi$	132.71(5)	$^{229}Rn \rightarrow {}^{215}Po$	125.7(16)
4	$^{213}Po \rightarrow {}^{209}Pb$	158.79(6)	$^{215}Po \rightarrow {}^{211}Pb$	140.11(3)

Table 1. Mean alpha-decay recoil energies of the first four alpha-decay progeny in the ^{225}Ac and ^{227}Ac decay chains.

Nuclear recoil following beta decay was considered to be negligible. Each simulation layer corresponds to a single transition between states shown in fig. 1 in the main text. The simulated material dimensions were selected for each decay to be between a factor 3 and 10 greater than the daughter recoil range, such that no particles escaped the system in a direction perpendicular to the foil depth, while at the same time ensuring that the simulation accounted for monolayer-scale interactions, thus increasing accuracy. For the first decay, a direction according to isotropic emission was sampled for each implanted particle. The daughter nucleus was then made to recoil along this direction with its corresponding recoil energy. The number of particles that were retained on the foil were recorded each time, while the number that 'backscattered' i.e., were ejected in the direction of the PIPS detector by virtue of their recoil were also recorded. The backscatter file contained information of the particle energy immediately after leaving the foil surface, as well as its normalized momentum projected along the x, y and z axes. In order to determine the implanted distribution of backscattered recoil daughters in the detector, the lateral positions on the sample foil were re-sampled from a Gaussian distribution of standard deviations of 0.5 cm, reflecting the size of the implanted beam. From each sampled starting position, it was determined in advance if the recoil would be incident on the detector by calculating the emission angle and comparing it to that subtending the 9.77mm radius detector surface at the corresponding sample-detector distance of 7 mm, 13.5 mm and 9 mm for samples A, B and C respectively. If it was projected that the recoiling daughter would be incident on the detector it was kept in the simulation, whereas all those whose emission angles were too large to be incident on the detector were discarded from the simulation. The lateral positions of the particles determined to be incident on the detector were then once again collapsed to 0, and implantation was simulated in a Si-disk (detector) on a microscopic scale. As before, this allowed the precise depth distribution of the recoiling daughters in the detector to be determined. The output of this step in the simulation were the end-point positions of alpha-decay recoil daughters in the Si disk and the Al sample foil. The end-point positions of further alpha-decay progeny up to the third alpha-decay generation were similarly modelled from the end-point positions of their parents, accounting for ejections from sample foil to disk in each step.
At first, Only foil-detector and detector-detector transitions were considered. However, it was later observed that the calculated geometric efficiencies led to a systematic difference in calculated ^{225}Ac activities from its different daughter nuclides. As a consequence, simulation steps for detector - foil transitions were performed to accounting for the 'ping-pong' effect that was initially considered negligible. It was observed that the calculated activities from the 'ping-pong-corrected' geometric efficiencies were closer and the systematic differences were much smaller than without accounting for this effect.
The upper panels in fig. 7 show the position distributions for ^{225}Ac and ^{227}Ac progeny for each of the samples. The difference in the distributions between samples is due to different implantation energies and/or sample-detector distances used for the

alpha spectrometry studies. The total geometric efficiencies for the ith generation alpha decay daughter of implanted parent isotope X_0 were calculated from eq. (1).

$$\varepsilon_{geo}(X_i) = n_d(X_i)\varepsilon_\alpha^{d\to d} + n_f(X_i)\varepsilon_\alpha^{f\to d} + n_{fdf}(X_i)\varepsilon_\alpha^{fdf\to d} \tag{1}$$

In this equation, n_d, n_f, and n_{fdf} refer to the relative population of atoms that recoiled from the foil to detector, remained on the foil, or 'ping-ponged' from foil to detector and back to foil respectively. n_d also encompasses the population that has 'double ping-ponged', i.e. recoiled from foil to detector, from detector to foil, and again from the foil to detector. This is only relevant for third generation alpha recoil daughters and further progeny. The corresponding geometric efficiencies for alpha particle detection from each position are also present in the equation. $\varepsilon_\alpha^{d\to d} = 50$ % is the geometric efficiency for alpha detection in the detector from a nuclide on the detector. An absolute error of 2% is attributed to this number as backscattering of the alpha particle can occur leading to deviations from 50% efficiency depending on the decaying nuclide's implantation depth in the PIPS detector. The geometric term, $\varepsilon_\alpha^{f\to d}$, was taken to be equal to that of a point source at the associated source-detector distance. A measurement error of 0.2 mm on the sample-detector distance was taken and in each case propagated to the geometric efficiency error. Finally, the term $\varepsilon_\alpha^{fdf\to d}$ refers to the alpha detection efficiency for nuclides that have ping ponged from the detector back to the sample foil. The macroscopic surface distribution of ping-ponged progeny on the sample foil is vastly different from the point source, so $\varepsilon_\alpha^{fdf\to d}$ was calculated by sampling isotropic emission from the ping-ponged surface distribution and counting the fraction of alpha particles incident on the detector surface. In the cases where a calculated contribution to the geometric efficiency depended on a number of simulated particles, i.e. the terms, $n_d(X)$, $n_f(X)$, $n_fdf(X)$, and $\varepsilon_\alpha^{fdf\to d}$, the error was taken be the standard deviation of the Poisson distribution with mean corresponding to the associated simulated value.

The quantities $\varepsilon_{X_i}^{f\to d}(X_0)$ in eq. (4) in the main text used for the alpha spectrometry of recoil daughters (α-srp) technique were recovered from the simulation through counting the number of atoms, $n_d(X_i)$ remaining on the detector after the ith simulation layer.

Simulations were carried for all three samples for ^{225}Ac and ^{227}Ac with the ping-pong effect neglected, while the ping-pong correction simulations were only performed for the ^{225}Ac chain. The resulting geometric efficiencies are displayed in the bottom panels of fig. 7. For all distances, the geometric efficiency of daughters of ^{225}Ac and ^{227}Ac is similar with a growing relative deviation with each subsequent alpha decay generation that reaches up to approximately 3% by generation 3 for the 7 mm source detector distance. The importance of the ping-pong correction was most important for the closest (7 mm) source-detector distance, leading to a relative 6% higher geometric efficiency for ^{213}Po.

3 Count rate histograms for alpha-decay recoil spectrometry

In section 4 in the main text, the α-srp technique is described. Here, count rate histograms of ^{217}At, ^{219}Rn and ^{215}Po that were used to calculate the end of implantation (e.o.i) count rate of recoil daughters, $R_{X_i}(t_{out})$, are shown. For completeness, histograms are also shown for count rates gated on ^{213}Po for each sample, as well as the count rates gated on ^{214}Po and ^{218}Po alpha decay peaks that were identified in sample A. The time spectra were constructed by energy-gating on the prominent alpha-decay energy peaks shown in fig. 2 in the main text. The spectra were fit with an unconstrained exponential function, maximizing the log-likelihood objective function with Poisson-errors on each bin. The fitted e.o.i count rate parameter in the legend of each subfigure shown in fig. 8 are displayed in table 2 in the main text. The decay constant of each fit corresponds to that of its longest lived decay parent implanted in the detector.

4 Sample activity measurements

4.1 Alpha decay spectrometry of ^{225}Ac

The alpha spectrometry was performed for each sample in the 'alpha setup decay spectroscopy chamber' (ASET) with a 300 mm^2 active area partially depleted passivated implanted planar silicon (PIPS) detector for all measurements. The samples were mounted on a movable sample holder (ladder) that was aligned with the detector. Pulse timing and amplitude of events registered by the detector were processed by a CAEN N6724s pulse height analysis digitizer and stored on a computer for further analysis. For each of the samples, the count rates of nuclide X, $R^{(X)}(t)$ of the 6341, 6241 and 6126 keV peaks of ^{221}Fr, the 7066.9 keV peak of ^{217}At and the 8376 keV peak of ^{213}Po were taken for analysis from the alpha decay energy spectrum. ^{225}Ac and ^{213}Bi peaks were not used as they overlapped with each other. ^{225}Ac activities were calculated from each nuclide analyzed according to eq. (2).

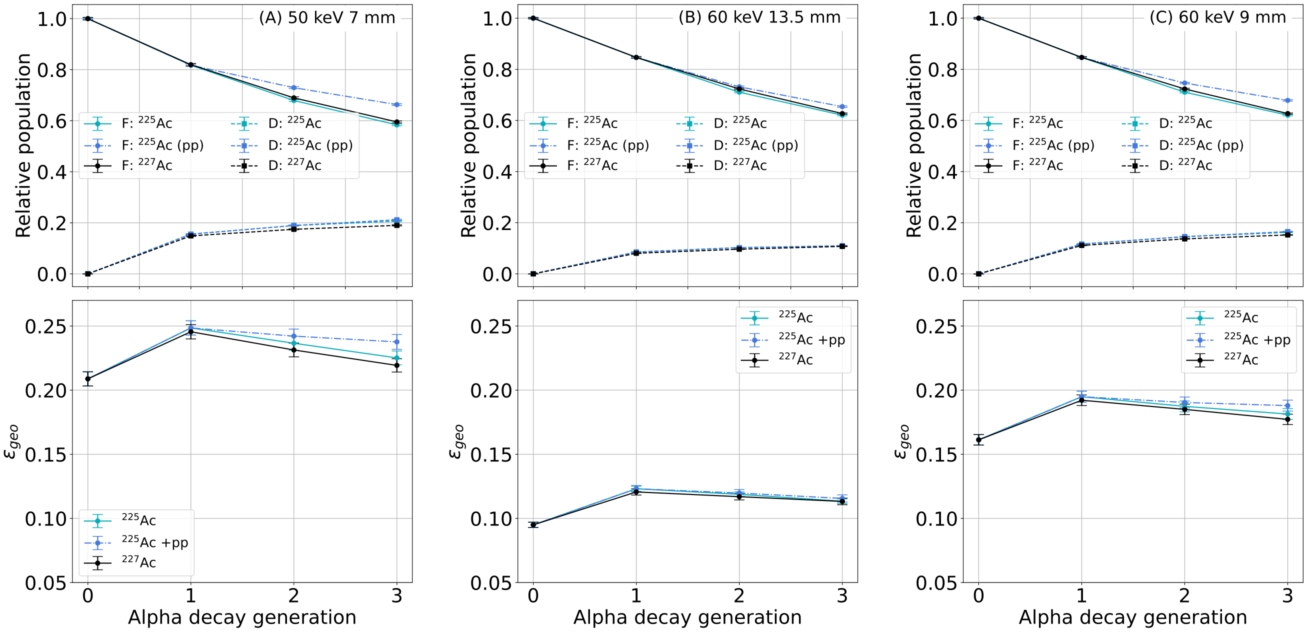

(a) Sample A **(b)** Sample B **(c)** Sample C

Figure 7. Above: The relative distributions of alpha decay daughters of the ^{227}Ac and ^{225}Ac decay chains between the sample foil (F) and PIPS detector (D) surface during α spectrometry. The dashed-dotted lines represent the cases where the ping-pong (pp) effect of the ^{225}Acdecay chain is accounted for. Below: The corresponding alpha particle detection efficiencies calculated with eq. (1).

$$A^{(B)}(t) = \frac{R^{(X)}(t)}{\varepsilon_{geo}^{(X)} \mathscr{F}_B^{(X)} (1 - R_{tot}\tau)} \quad (2)$$

Here $R^{(X)}(t)$ represents the alpha particle count rate of alpha-decay line X, $(1 - R_{tot}\tau)$ is the detector live-time fraction, with R_{tot} the count rate of all recorded events, and τ the system dead-time. $\mathscr{F}_B^{(X)}$ is the fraction of decays of ^{225}Ac that lead to alpha decay X, accounting for branching ratios and the ratio of ^{225}Ac daughter nuclide activity to that of ^{225}Ac in secular equilibrium. $\varepsilon_{geo}^{(X)}$ is the geometric efficiency for detection of an alpha particle of alpha-decay line X that is isotope dependent due to redistribution through alpha-decay recoil.

For samples A and C, the count rates of ^{221}Fr, ^{217}At and ^{213}Po were obtained by integrating the corresponding peaks in the energy spectrum and dividing by the measurement time.

The count rates of ^{221}Fr, ^{217}At and ^{213}Po in sample B were obtained using a different method due to an issue with the digitizer parameter set. The trapezoid shaping algorithm that allowed a channel to be assigned to a given input signal height was set such that a pulse recorded between 5 and 45 µs after the previous one was offset in the spectrum by several hundred channels. 'Ghost' peaks were visible in the energy spectrum and counts that should have been registered in the region of interest were registered at higher energies in the spectrum. To calculate the true count rate, spectra of time difference between an event gated on the ^{221}Fr ^{217}At and ^{213}Po peak regions respectively and any previous event were plotted. Data occurring at a time difference between 5 and 45 us were removed, and the resulting spectrum was fitted with an exponential decay function. The integral of the fitted function was then used to determine the true count rate for each peak.

To determine the live-time fraction, the total count rate in the PIPS detector, $R_{tot}(t)$ was first obtained from each spectrum by integrating all counts and dividing by the true measurement time. The dead-time parameter was taken to be 1.29 (20) µs calculated in previous work for the same detector and digitizer set using the decaying source method[2,3]. It was observed that the calculated activities were strongly insensitive to the precise value of the dead-time parameter due to count rates of approximately three orders of magnitude less than the inverse of the dead-time parameter. The Bateman factors for ^{225}Ac daughters in secular equilibrium with ^{225}Ac were calculated assuming branching ratios of 1 in the decay chains. Values of 1.000345, 1.000345, 1.00355 were obtained for ^{221}Fr, ^{217}At and ^{213}Po respectively. These were then multiplied by the cumulative alpha-decay

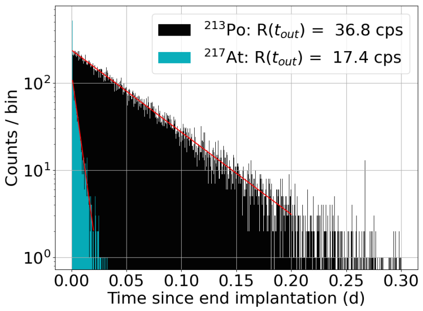

(a) Sample A: ^{225}Ac chain

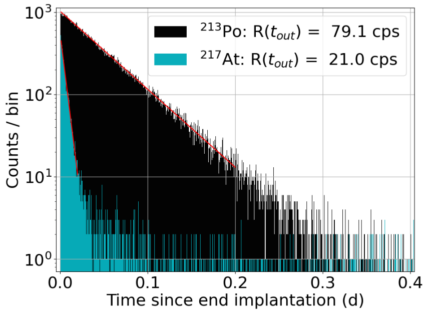

(b) Sample B: ^{225}Ac chain

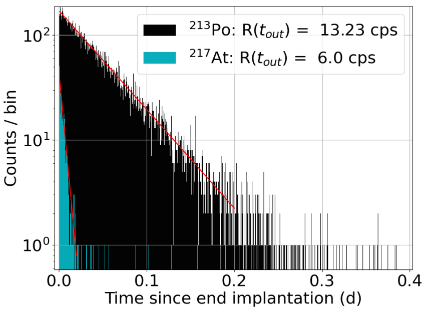

(c) Sample C: ^{225}Ac chain

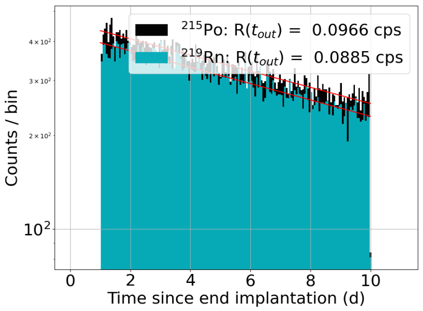

(d) Sample A: ^{227}Ac chain

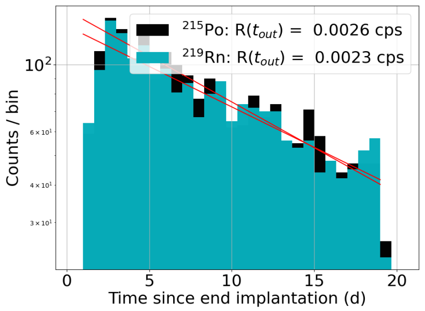

(e) Sample B: ^{227}Ac chain

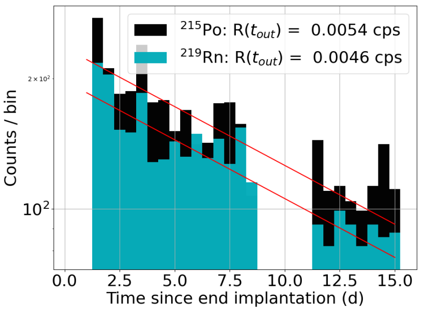

(f) Sample C: ^{227}Ac chain

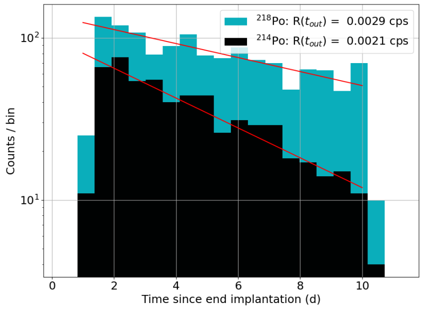

(g) Sample A: ^{226}Ra chain

Figure 8. Energy-gated decay time spectra for ^{225}Ac decay daughters (upper panels) ^{227}Ac decay daughters (mid panels), and ^{226}Ra decay daughters(lower panel), fit with exponential decay.

	E.o.c. activity						
	**Sample A**		**Sample B**		**Sample C**		
**Radiation**	^{225}Ac(kBq)	^{227}Ac(Bq)	^{225}Ac(kBq)	^{225}Ra(kBq)	^{225}Ac(kBq)	^{225}Ra(kBq)	^{227}Ac(Bq)
^{221}Fr α	57(4)		77(2)	11(2)	95(7)	0*	
^{217}At α	57(4)		80(2)	11(2)	97(7)	0*	
^{213}Po α	59(4)		81(2)	11(2)	99(7)	0*	
^{221}Fr γ 218 keV	57(4)				98(7)	0*	
^{213}Bi γ 440 keV	51(2)				88(3)	0*	
^{209}Tl γ 465 keV	49(5)				85(6)	0*	
^{209}Tl γ 1567 keV	51(2)				84(7)	0*	
^{209}Tl $\gamma\gamma$ 465/1567 keV			103(47)	30(20)			
^{219}Rn α		3.70(35)					0.25(2)
^{215}Po α		3.91(37)					0.25(2)
**Final**	52.9(12)	3.70(25)	79.0(10)	11.3(12)	91.2(21)	0	0.25(2)

Table 2. Calculated end of collection activities from each analyzed decay radiation for ^{225}Ac ^{225}Ra and ^{227}Ac progeny. Only activities calculated by direct decay spectrometry means are included. *Value consistent with 0 within error.

branching ratios to recover the Bateman decay and branching term, $\mathscr{F}_B$. In the samples where ^{225}Ra feeding was present, the Bateman decay factors are time dependent up to a time of approximately 100 days at which secular equilibrium of ^{225}Ac daughters is reached with ^{225}Ac, however due to the small values concerned (less than 0.1% of the ^{225}Ac activity), this time dependence was not taken into account. The live-time corrected alpha decay activities of the daughter nuclides that were calculated were finally converted to the ^{225}Ac activity on the foil by division of the appropriate alpha decay branching ratios. The output of this analysis was a time-series of ^{225}Ac activity data points that was fitted to obtain the end of collection (e.o.c.) activity of ^{225}Ac for all samples and ^{225}Ra for samples B and C.

For sample A, a fit according to the radioactive decay law was performed. For samples B and C, the presence of ^{225}Ra was accounted for by fitting the calculated ^{225}Ac activity with eq. (7) in the main text. In the case of sample A, the fit parameter of the ^{225}Ac decay constant (experimental value 6.986 (2) $\times 10^{-2}$ d^{-1}) was constrained between 6.93 $\times 10^{-2}$ d^{-1} and 7.07 $\times 10^{-2}$ d^{-1}. For sample B more stringent limit constraints were put on the ^{225}Ac decay constant of 6.980 d^{-1} and 6.994 d^{-1} as lower and upper bounds respectively. The ^{225}Ra decay constant (experimental value 4.65 (6) $\times 10^{-2}$ d^{-1} was constrained between 4.47 $\times 10^{-2}$ d^{-1} and 4.95 $\times 10^{-2}$ d^{-1}. These measures was necessary as the correlation between fitted ^{225}Ac and ^{225}Ra decay constants and ^{225}Ra and ^{225}Ac e.o.c. activities were observed to be high. The constraints thus enabled the e.o.c. activities to be more accurately determined. Finally, for sample C, the lower and upper bound constraints for the ^{225}Ac decay constant were 6.92 $\times 10^{-2}$ d^{-1} and 7.06 $\times 10^{-2}$ d^{-1}, while those for the ^{225}Ra decay constant were 3.72 $\times 10^{-2}$ d^{-1} and 5.58 $\times 10^{-2}$ d^{-1}.

The alpha decay curves are shown in fig. 9 along with the relative statistical residuals between the fitted functions and the data points. The above panels show that decay curves that were used for determining the e.o.c. activities of ^{225}Ac and ^{225}Ra. For samples A and C, the activities from each of the analyzed nuclides show very good agreement. This is only true when the ping-pong effect is accounted for. As shown in the lower panels of fig. 9 where the ping-pong effect is neglected, the deviation in calculated ^{225}Ac activities from each of the analyzed nuclides show larger systematic shifts. The final calculated values of ^{225}Ac and ^{225}Ra e.o.c. activities are given in table 2

4.2 Alpha decay spectrometry after long waiting time

In order to validate the recoil decay spectrometry methods described in the main text of this work, alpha spectrometry was performed on samples A and C 297 and 276 days after e.o.c. respectively. The alpha spectra are shown in fig. 10. Each sample was placed a distance of 9 mm from the detector. Count rates from prominent peaks of ^{215}Po and ^{219}Rn were analyzed for calculation of the ^{227}Ac activity.

The point-source geometric efficiency, $\varepsilon^{\circ}_{geo}$ was initially determined by measuring the alpha-decay count rate on the PIPS detector of a triple alpha (^{239}Pu, ^{241}Am, ^{244}Cm) calibration source placed in the sample mount. It was calculated as the mean of the count rate from each nuclide in the source as a fraction of its activity with a 10% nominal uncertainty. As discussed in section 2, an alpha decay generation-dependent geometric efficiency is however necessary to obtain accurate activities from the measured count rates of the ^{227}Ac daughters. The geometric efficiencies for the analyzed peaks were calculated by correcting for the recoil dynamics. The corrected recoil efficiency for nuclide X, $\varepsilon^{'}_{geo}(X)$ for the standard alpha spectrometry of the ^{227}Ac chain was calculated from eq. (3), with $\varepsilon_{geo}(X)$ the geometric efficiency of nuclide X in the ^{227}Ac chain at 9mm distance as shown in fig. 7c and $\varepsilon_{geo}(^{227}Ac)$, the geometric efficiency of ^{227}Ac calculated for a point source at 9mm source-detector

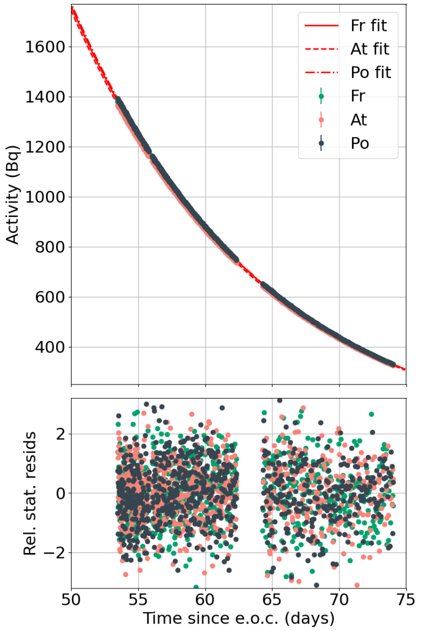

(a) Sample A

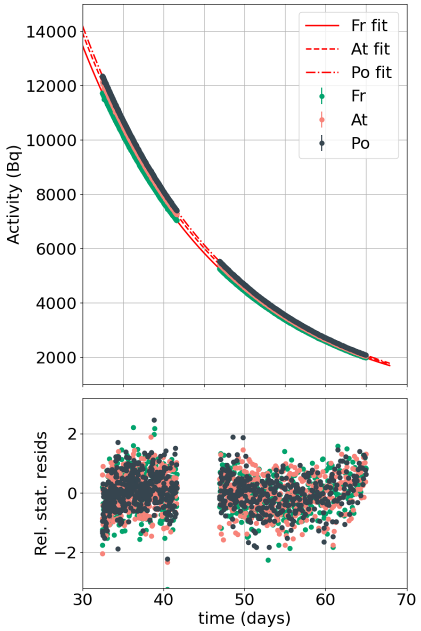

(b) Sample B

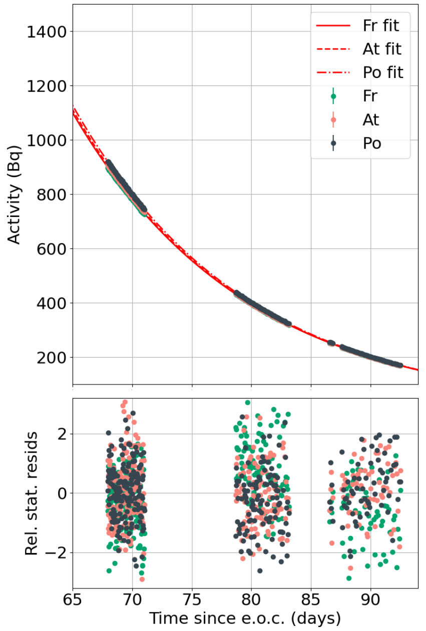

(c) Sample C

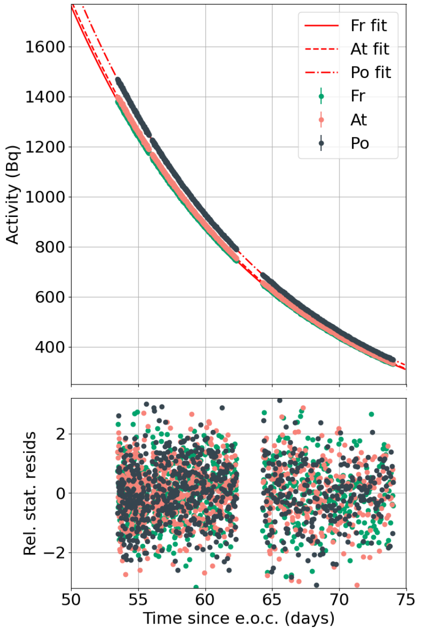

(d) Sample A

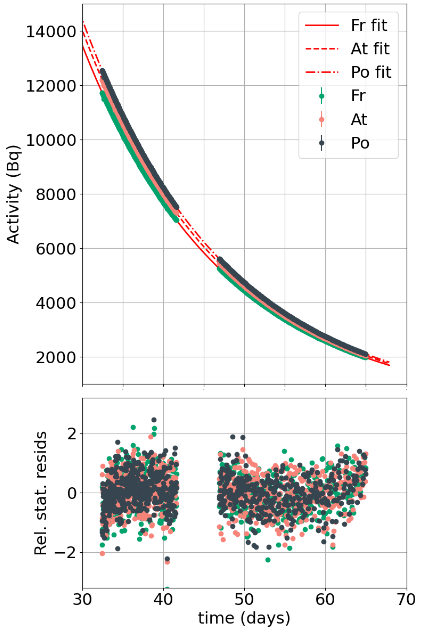

(e) Sample B

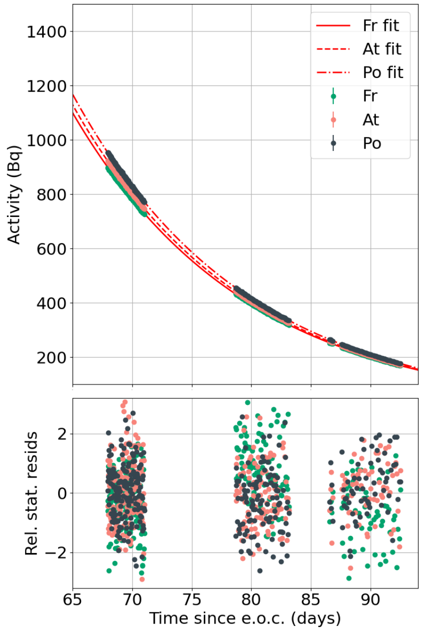

(f) Sample C

Figure 9. Alpha decay activities of ^{225}Ac from ^{221}Fr, ^{217}At and ^{213}Po alpha decays. Upper panels: Activities calculated with geometric efficiencies with ‘ping-pong’ correction. Lower panels: Activities calculated with geometric efficiencies neglecting ping-pong corrections.

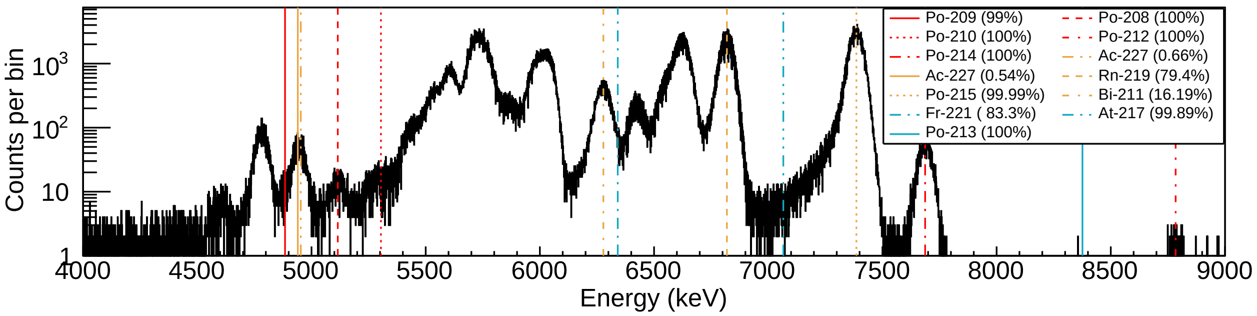

(a) Sample A

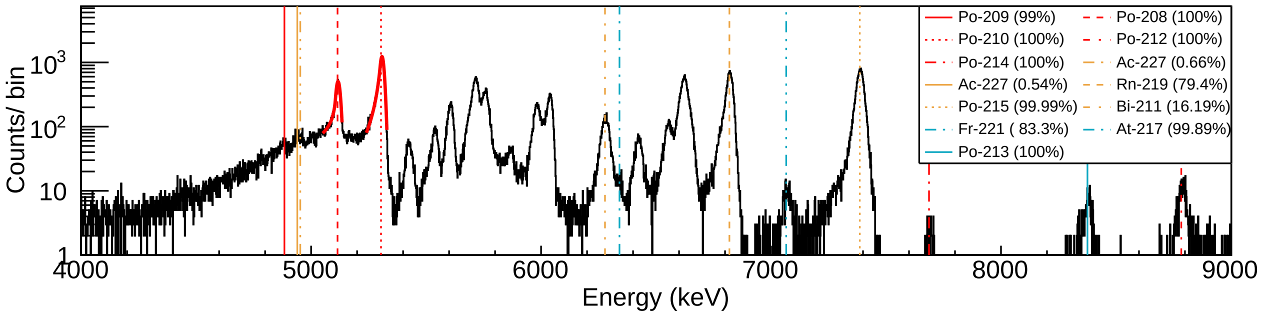

(b) Sample C

Figure 10. Alpha decay spectra of samples A and C after 297 and 276 days since e.o.c respectively. Alpha decay branching ratios indicated in legend. ^{219}Rn and ^{215}Po lines were analyzed for ^{227}Ac activity calculation. Peaks of ^{208}Po,^{210}Po,^{212}Po and ^{214}Po were analyzed as described in the text.

distance. A correction factor to account for the ping-pong effect for isotope X was taken to be the ratio of geometric efficiencies with and without this effect calculated for the equivalent alpha decay generation daughter in the ^{225}Ac decay chain, $\frac{\varepsilon^{pp}_{geo}(\tilde{X})}{\varepsilon^{npp}_{geo}(\tilde{X})}$.

$$\varepsilon'_{geo}(X) = \varepsilon^{\circ}_{geo} \frac{\varepsilon^{pp}_{geo}(\tilde{X})}{\varepsilon^{npp}_{geo}(\tilde{X})} \frac{\varepsilon_{geo}(X)}{\varepsilon_{geo}(^{227}Ac)} \tag{3}$$

The e.o.c. ^{227}Ac activities were calculated from the measured ^{219}Rn, and ^{215}Po activities at the time of measurement by evaluating the Bateman equation at these times. The final activities are given in table 2.

Several other nuclides were identified in the alpha decay spectra in fig. 10, that were analyzed for sample C. Peaks with relatively high count rate at 5115 keV and 5304 keV are due to ^{208}Po and ^{210}Po respectively. Counts were also observed that corresponded to the ^{214}Po and ^{212}Po alpha decay energies of 7687 keV and 8784 keV respectively. Most other peaks in the 5400 - 6800 keV region come from the ^{225}Ac decay chain.
E.o.c. activities were calculated for each of the above mentioned Po isotopes. The counts due to ^{214}Po and ^{212}Po were integrated and divided by the measurement time to obtain the count rate. This was converted to an activity by considering the geometric efficiency to be the same as for ^{215}Po in the ^{225}Ac decay chain (i.e. accounting for the recoil dynamics as these nuclides were considered to be present due to implantation of precursor nuclides).
Alpha energy peaks of ^{208}Po and ^{210}Po were each fitted with a crystal ball function[4] due to overlap with alpha counts from other peaks. The crystal ball function describes the alpha energy line-shape as a Gaussian, parameterized by amplitude, C, centroid, x_0, standard deviation, σ, with an exponential tail parameterized by the tail onset parameter, α, and the tailing degree parameter, n. The integral of the crystal ball function was calculated and evaluated for the fitted parameters. The fitted tailing degree for the ^{208}Po peak was < 1, so the integral was calculated in the range $(x_0 - 10\alpha\sigma, \infty]$ to avoid divergence. The activity at measurement time was then calculated from the resulting count rate assuming the point source geometric efficiency at 9 cm. The calculated activities are given in table 3.
The calculated activity at the time of the spectrometry was decay-corrected to e.o.c. activity for ^{208}Po. For ^{212}Po and ^{214}Po, the e.o.c. activity of the precursor nuclide was calculated using the Bateman equation. Finally, the e.o.c. activity of ^{210}Po was deduced through first calculating the contribution to its measured activity from the e.o.c. activity of ^{226}Ac, calculated from the measured ^{214}Po activity. This was then subtracted from the measured ^{210}Po activity. The resulting uncertainty was more than 100%, therefore it is considered that no directly implanted ^{210}Po was detected. The e.o.c. activity of each of the precursor nuclides in the final column table 3 was compared to its in-target production yield, shown in table 1 in the main text. These values were used to calculate the separation enhancement factors of ^{225}Ac relative to the analyzed nuclides that are presented in table 3 in the main text.

Finally, in this analysis a minimum detectable activity was determined for ^{209}Po ($T_{1/2}$ = 124 Y). The background in the energy window 4860 keV - 4910 keV corresponding to the ^{209}Po alpha decay energies of 4883 keV and 4885 keV was determined through linear interpolation of the tailing from the ^{208}Po and ^{210}Po peaks. This was used as input for the Currie equation where the MDA to a 95% confidence interval was determined to be 2.32(7) $\times 10^{-3}$ Bq, assuming a point source efficiency at 9 mm.

Nuclide	Method	Dec. Lines. (keV)	Activity (Bq)	Precurs.	$A_{e.o.c.}$. precurs. (Bq)
^{206}Po	γ	1032, 286, 522, 980, 881, 1718, 537	$9.8(4) \times 10^{2*}$	^{206}PoO	$9.8(4) \times 10^{2}$
^{208}Po	α_{long}	5115	$1.04(4) \times 10^{-1}$	^{208}PoO	$1.25(5) \times 10^{-1}$
^{210}Po	α_{long}	5304	$2.23(7) \times 10^{-1}$	^{210}PoO, ^{226}Ac	$0^{\dagger}$, $2.2(3) \times 10^{3\ddagger}$
^{212}Po	α_{long}	8784	$5.6(4) \times 10^{-3}$	^{228}Ac	$3.2(2) \times 10^{1}$
^{214}Po	α_{long}	7687	$7.7(10) \times 10^{-4}$	^{226}Ac	$2.2(3) \times 10^{3}$

Table 3. Other nuclides identified in sample C, from either alpha-decay spectrometry at 276 d after e.o.c., or during the gamma-ray spectrometry measurements. Analyzed decay lines are given in the third column. Quoted activities measured by alpha-decay spectrometry are given at measurement time, while the activity of ^{206}Po is decay corrected to e.o.c., as shown in fig. 13a (denoted by *). Precursor nuclides were deduced to not be due to implanted Ra nuclides through the absence of ^{225}Ra measured in sample C.
†: Value consistent with 0.
‡: deduced from ^{214}Po as described in the text.

4.3 Gamma-ray spectrometry

4.3.1 ^{225}Ac

The gamma-ray spectrometry was performed in a lead castle with a Canberra high purity germanium (HPGe) detector. In each campaign, the sample vial was placed at a distance 10 cm from the HPGe detector. The measurement campaigns for samples A and C lasted for a total of 11 days and 17 days respectively. Neither source was moved from the setup during the campaigns. The gamma lines analyzed were the 218, 440, 465 and 1567 keV decays of ^{221}Fr, ^{213}Bi, ^{209}Tl, ^{213}Bi, and ^{209}Tl respectively. The count rate of each of the peaks, $R^{(X)}(t)$ was determined from integrating the Compton-background subtracted spectra and dividing by the real measurement time. The Background subtraction was performed using a method based on the Sensitive Nonlinear Iterative Peak (SNIP) clipping algorithm, implemented in ROOT[5,6]. Measurements typically lasted between 8 and 16 hours. The associated timestamps, t, correspond to the median measurement time. The activity of ^{225}Ac at each measurement time, $A^{(B)}(t)$, was calculated using eq. (4), with X representing the particular gamma line analyzed, I_γ representing the relative gamma decay intensity per parent decay, $\mathscr{F}_B^{(X)}$ representing the Bateman factor of the nuclide with gamma line X with respect to ^{225}Ac in secular equilibrium, and $\varepsilon_{tot}^{(X)}$ representing the total detector efficiency at the energy of the analyzed gamma line of X.

$$A^{(B)}(t) = \frac{R^{(X)}(t)}{I_\gamma^{(X)} \mathscr{F}_B^{(X)} \varepsilon_{tot}^{(X)}} \tag{4}$$

The major source of uncertainty on the final ^{225}Ac activities were the systematic errors on the detector efficiency at different energies. This is manifest in fig. 12 as the uncertainty bands dominate the statistical scatter of data points. Due to the detector efficiency, a systematic offset for the activities calculated from the ^{221}Fr 218 keV gamma line is observed with respect to the rest of the analyzed gamma lines.
For analysis of sample A, a Monte-Carlo fitting approach was taken to account for both the systematic and statistically uncertainties on the calculated activities. For each Monte-Carlo procedure, the ^{225}Ac activity data points were resampled by dividing the corresponding count rate by values of branching ratios and total detection efficiency drawn from their respective Gaussian probability distributions with standard deviations given by their systematic uncertainties. Fits were then performed for each Monte-Carlo run minimizing the chi-squared objective function. As no ^{225}Ra was present in this sample, a simple exponential fit function was used, with the e.o.c. ^{225}Ac activity being the only free parameter. The decay constant was fixed to that of ^{225}Ac. The fitted curves in fig. 11a are those parameterized by the Mean ^{225}Ac e.o.c. activities from the Monte Carlo fitting, while the shaded regions correspond to those parameterized by the mean $\pm$ 1 standard deviations of the e.o.c. ^{225}Ac activity distribution.
For Sample C, the data was fit with eq. (7) due to the possible presence of ^{225}Ra, with a chi-squared minimization of the activity data with the statistical uncertainty. The shaded band corresponds to the fitted curve multiplied by the $\pm$ 1 σ relative systematic uncertainty values due to detector efficiency, branching ratios and gamma intensities added in quadrature for each analyzed line. The e.o.c ^{225}Ac and ^{225}Ra activities in the sample were calculated as the weighted mean of the activities from each of the analyzed gamma lines. The resulting values for each gamma line followed are summarized in the appropriate row of table 2.

4.3.2 ^{226}Ra

In addition to gamma-decay lines identified from ^{225}Ac and daughter decays, gamma-decay lines of other nuclides were identified. Firstly, several gamma-decay lines for daughter peaks of ^{226}Ra were analyzed. For sample A, the 351.9 and 487.1 keV peaks of ^{214}Pb were analyzed, along with the 609.3, 1764.5, 1120.3, 1238.1, 2204.1, 578.4, 1377.7 and 934.1 keV peaks of ^{214}Bi. The same peaks were analyzed for sample C with the exception of the 2204.1 keV peak that was not observed due to pre-amplifier gain settings. As ^{226}Ra is present in the radioactive background from the ^{nat}U decay chain, a long background measurement of 4.8 d was taken. This background count rate of ^{226}Ra was subtracted from the count rate of each of the

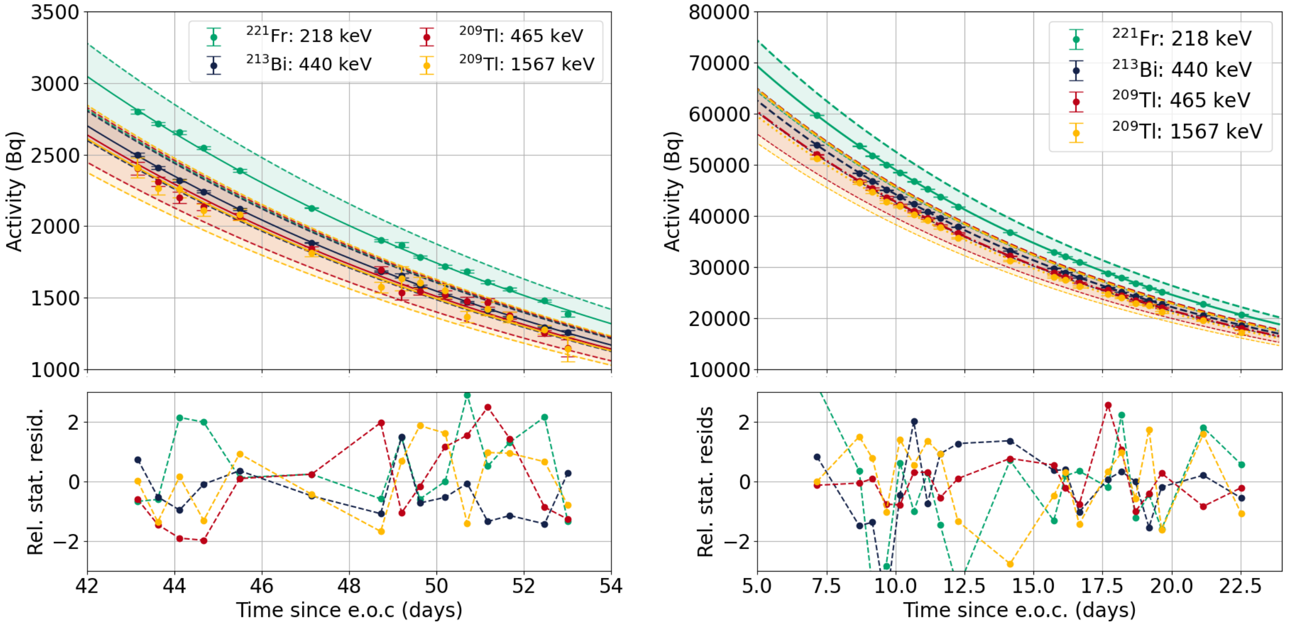

(a) Sample A **(b)** Sample C

Figure 11. Gamma spectrometry activity curves of samples A and C. The top panels show decay data with the colored band representing the systematic uncertainty due to detector efficiency calibration, nuclear data branching ratios and gamma decay intensities. The bottom panels show the relative statistical residuals.

analyzed peaks. The activity was then calculated from the background-subtracted count rate of each analyzed gamma line divided by the gamma decay intensity, branching ratios, and total detector efficiency at the appropriate energy. The resulting data are plotted in fig. 12 as the activity of each analyzed gamma line relative to background.

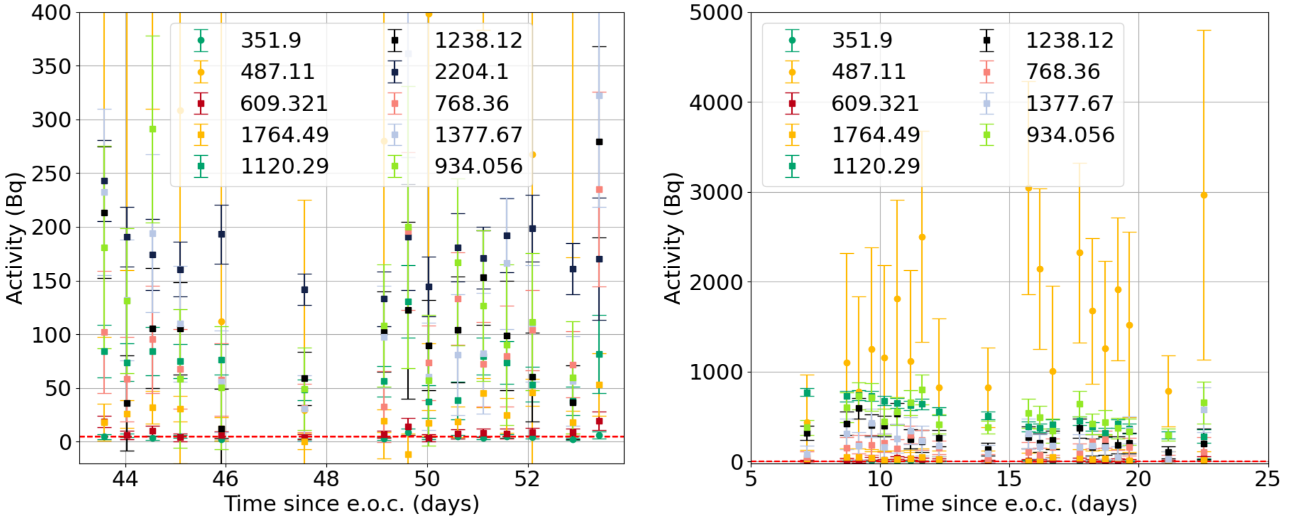

(a) Sample A **(b)** Sample C

Figure 12. Gamma spectrometry activities of ^{226}Ra. The relative statistical uncertainty of the 351.9 keV line of ^{214}Pb was an order of magnitude lower than almost all other lines meaning it gave the highest statistical weight to the calculated mean activity

The resulting ^{226}Ra activities were dominated by the background-subtracted ^{214}Pb peak at 351.9 keV due to a combination of its high gamma intensity (35.72 %) and relatively higher detector efficiency at this energy. The weighted mean ^{226}Ra activity in sample A and C was 4.7(4) and 4.1 (7) Bq respectively. These values contrast with the ^{226}Ra activities derived through alpha

decay spectrometry. A possible reason for the discrepancy is the changes in background radiation, that were not monitored at the time of the measurement campaign. For this reason, these calculated values were rejected from further analysis.

4.3.3 ^{206}Po

Another radioactive decay chain was identified in the gamma-decay spectrum of sample C, originating from ^{206}Po. Several gamma-decay lines from decay of this nuclide as well as its beta-decay daughter nuclide ^{206}Bi were identified. ^{206}Po peaks with gamma decay energies of 1032.26, 286.41, 522.47 and 980.23 keV were analyzed, while the 881, 1718 and 537.45 keV peaks of ^{206}Bi were analyzed. Figure 13a shows the plotted activity data for each of the peaks.

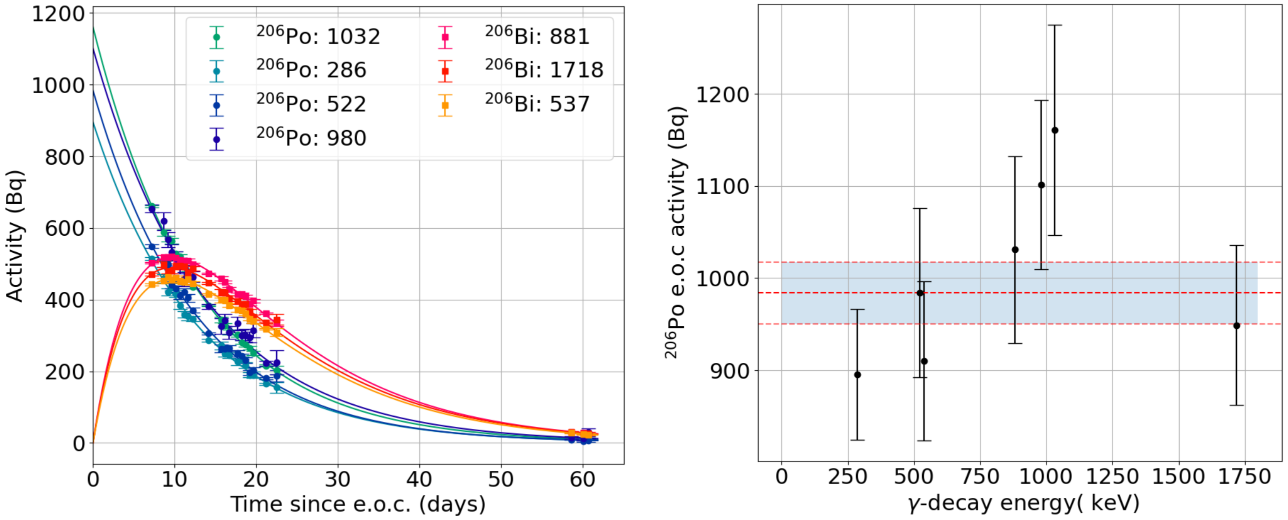

(a) Sample C decay curves

(b) Sample C mean e.o.c. activity

Figure 13. Gamma spectrometry activities of ^{206}Po and daughter ^{206}Bi.

The activities of the analyzed nuclides were each calculated using eq. (4) with $\mathscr{F}_B = 1$. The activity data of ^{206}Po was fit with the standard radioactive decay equation, while the decay data of ^{206}Bi was fit with eq. (5), meaning that the ^{206}Bi was produced uniquely from feeding from ^{206}Po.

$$A^{(B)}(t) = A^{(A)}_{e.o.c} \frac{\lambda_B}{\lambda_B - \lambda_A} \left(e^{-\lambda_A t} - e^{-\lambda_B t} \right) \tag{5}$$

In each fitting procedure, the decay constant of the nuclides were allowed to vary by a maximum of 10% from their values taken from ENSDF. The excellent fits confirm that ^{206}Po was implanted and that the observed ^{206}Bi was generated by decay-feeding. Figure 13b shows the derived e.o.c activity of ^{206}Po derived from the analysis of each gamma-decay line. A weighted mean activity of 984(34) Bq was calculated.
The presence of this nuclide in the sample is deduced to originate from implantation of ^{206}Po^{16}O^{+} as an ion beam of A/q = 222. Its presence is not detrimental for the use of mass-separated ^{225}Ac, as it can be radiochemically separated.

4.4 Gamma-gamma coincidence spectrometry

Gamma-gamma coincidence data was taken over a period of 10 d. The data was fitted with Monte-Carlo sampling of the data was performed. In each iteration, effective data-points were sampled from a Gaussian distribution of mean and standard deviation given by the measured data points and their 1σ statistical uncertainties respectively. The sampled data points were fitted with eq. (7) and the free parameters, $A_{e.o.c}(^{225}Ra)$ and$A_{e.o.c}(^{225}Ac)$ were recorded. This process was repeated 10000 times from which the mean and standard deviation of the e.o.c activities for ^{225}Ac and ^{225}Ra were derived to be 108(48) and 30(19) kBq respectively. This approach was first tried on test data and found to be much more reliable than a simple least-squares minimization fit of the data with eq. (7). The end fit result fixed with the mean values of $A_{e.o.c}(^{225}Ra)$ and $A_{e.o.c}(^{225}Ac)$, along with the 1σ error band from the individual $A_{e.o.c}(^{225}Ra)$ and$A_{e.o.c}(^{225}Ac)$ parameter distributions is shown in fig. 14.
The distribution of $A_{e.o.c}(^{225}Ra)$ and $A_{e.o.c}(^{225}Ac)$ values calculated from each Monte-Carlo fitting iteration are shown in the left-hand panel of fig. 14. The broadness of the distribution is due in part to the poor statistics of the $\gamma\gamma$ coincidences obtained

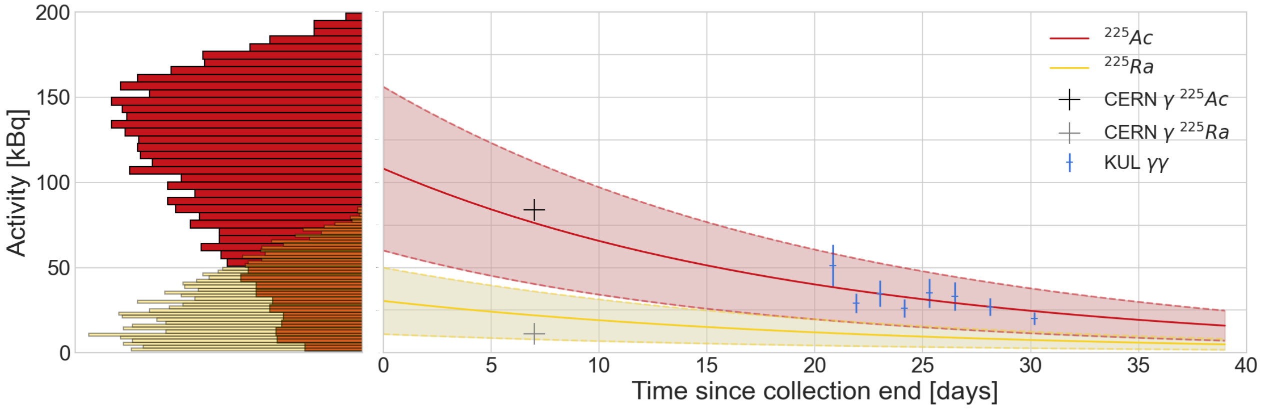

Figure 14. The activity of ^{225}Ac and ^{225}Ra as determined by $\gamma\gamma$ coincidence spectrometry. The red line represents the ^{225}Ac activity from both ^{225}Ac and feeding from ^{225}Ra present at the end of collection. The yellow line and band is the ^{225}Ra activity calculated from the fitted $A_{e.o.c}(^{225}Ra)$ parameter. The large error bands are due to the large amount of time elapsed from end of collection to measurement meaning a large range of end of collection activity values correspond with measured data within errors. The relatively high statistical errors and limited number of data points amplify this effect. $+$ denote activities measured by γ spectrometry at CERN before shipping the sample.

with the detectors far away along with the low branching ratio of the ^{225}Ac to ^{209}Tl through the 2.140% ^{213}Bi alpha-decay branch. Furthermore, due to the similarity of the ^{225}Ra and ^{225}Ac half-lives, the fitted resampled data led to values of e.o.c activities of ^{225}Ra and ^{225}Ac that were highly correlated. Finally, as the data were taken more than three weeks after the collection, they were less sensitive to the initial ^{225}Ra and ^{225}Ac activities. The final result is that the ^{225}Ac and ^{225}Ra activities could only be estimated with a very high statistical uncertainty, such that their weight in determining the mean e.o.c activities in combination with other decay spectrometry methods was small.

The gamma singles spectra acquired from measurement of this sample were also analyzed for presence of ^{226}Ra, that is produced through spallation in the ThO_2 material but may also be present as a material impurity - as suggested in section 3. The count rate of selected prominent gamma lines of ^{214}Pb and ^{214}Bi, the decay daughters of ^{226}Ra, were measured and converted to an equivalent ^{226}Ra activity. Background measurements using the detectors in the same configurations were performed, and the deduced background ^{226}Ra activity was subtracted from that calculated from the sample. The final result for each analyzed line is shown in fig. 15. A weighted mean of the analyzed gamma lines yielded an activity of 0.6(6) Bq, meaning negligible ^{226}Ra was detected in the sample.

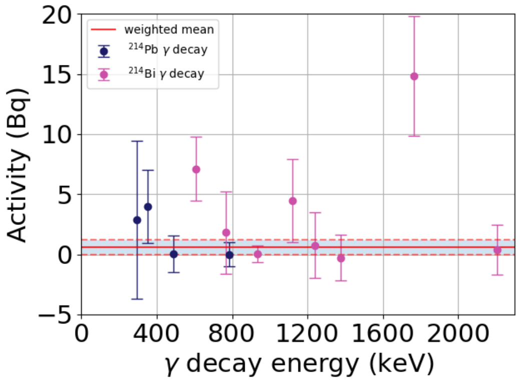

Figure 15. Activities of ^{226}Ra calculated from the progeny with gamma-decay energies shown in the figure. Red line is the mean activity of each analyzed gamma decay line. Grey band represents one-sigma confidence interval.

5 Sensitivity of α-srp for ^{227}Ac measurement

The measurement time needed for this technique to detect and quantify ^{227}Ac can be an order of magnitude more rapid than waiting for the ^{225}Ac to decay sufficiently to be able to observe and quantify ^{227}Ac peaks. The relative sensitivity of the technique to ^{223}Ra and daughters against ^{221}Fr and daughters has been determined to be approximately 10^4 times greater than direct alpha recoil spectrometry. The sensitivity analysis was performed by calculating the relative expected ^{223}Ra to ^{221}Fr count rate for different accumulation times, t_{acc} and implantation times, t_{imp}. The relative sensitivity of direct alpha spectrometry, σ_r^{dir} is given by eq. (6) while that of the α-srp technique, $\sigma_r^{\alpha-srp}$ is given by eq. (7).

$$\sigma_r^{dir} = \left(\prod_{i=1}^{2} \lambda_{Y_i}\right) \sum_{i=0}^{2} \frac{\exp(-\lambda_{Y_i} t)}{\prod_{j=0, j\neq i}^{2} (\lambda_{Y_j} - \lambda_{Y_i})} / \exp(-\lambda_{X_0} t) \tag{6}$$

$$\sigma_r^{\alpha-srp} = \frac{\mathscr{F}_B(t_{in}, t_{out}, Y_2)\, \varepsilon_{Y_2}^{f\to d}(Y_1)\, \varepsilon_\alpha^{d\to d}(Y_2) \exp(-\lambda_{Y_2}(t - t_{acc} - t_{imp}))}{\varepsilon_{X_1}^{f\to d}(X_0)\, \varepsilon_\alpha^{d\to d}(X_1) \exp(-\lambda_{X_1}(t - t_{acc} - t_{imp})) + f_{rss} \exp(-\lambda_{X_0} t)} \tag{7}$$

Here, Y references the ^{227}Ac decay chain with Y_0 = ^{227}Ac, Y_1 = ^{227}Th etc. Similarly, X references ^{225}Ac decay chain nuclides. The Bateman feeding term $\mathscr{F}$ and the geometric efficiency terms, ε are as defined in eq. (4) in the main text. f_{rss} is the recoil sputter yield of ^{225}Ac that contribute to ^{221}Fr alpha counts (i.e. the e.o.i. count rate of ^{221}Fr from recoil sputtering).
Both sensitivities are plotted in fig. 16 with $f_{rss} = 4(2) \times 10^{-6}$, corresponding to the mean value from the analysis of samples A and C respectively. $\varepsilon_{Y_2}^{f\to d}(Y_1)\varepsilon_\alpha^{d\to d}(Y_2) = \varepsilon_{X_1}^{f\to d}(X_0)\varepsilon_\alpha^{d\to d}(X_1) = 5.6(2)\%$ corresponding to the case for sample C.

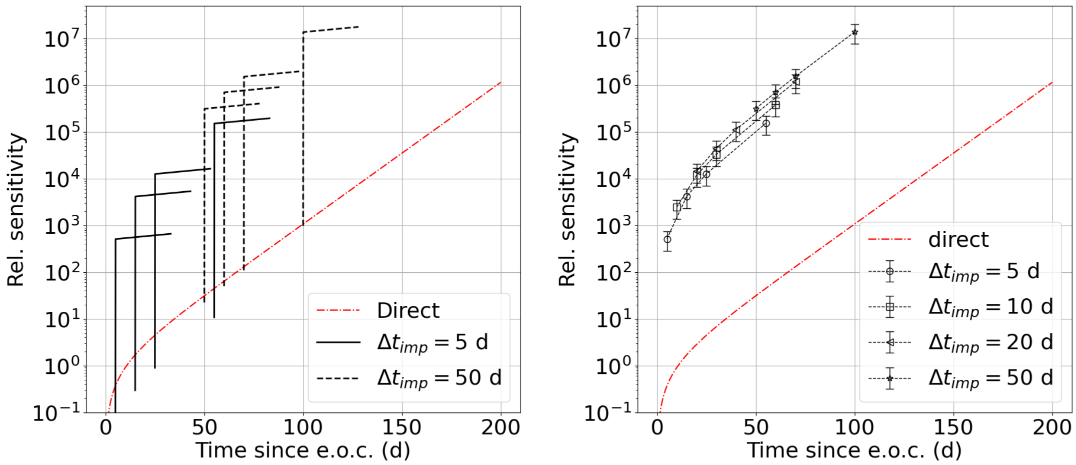

(a) Time dependence after e.o.i.

(b) 1 d after e.o.i.

Figure 16. Relative sensitivity of ^{223}Ra to ^{221}Fr detection for α-srp and direct alpha-decay spectrometry for accumulation times of $t_{acc} = 0$, 10, 20 and 50 d, and different implantation times, t_{imp}, shown in legend.

Figure 16a shows the results for 5 and 50 d implantation times, for accumulation times of 0, 10, 20 and 50 days respectively. The rapid increase in sensitivity at the end of implantation is due to the decay of ^{221}Fr, i.e. the first term in the denominator of eq. (7), that rapidly falls to 0. The sensitivity is therefore limited by the recoil-sputtered ^{225}Ac (and ping-pong grand-daughter activity). The sensitivity continues to increase after implantation due to the longer half-life of ^{223}Ra compared to ^{225}Ac. Figure 16b shows the sensitivity for the same waiting times, for four different implantation times, evaluated at 1-day after end of implantation. The gain in sensitivity remains in the 4 orders of magnitude range, with the highest sensitivity achieved for longer implantation times for any given time since e.o.c. ($t_{acc} + t_{imp}$). The orders of magnitude improvement in sensitivity could prove useful for quicker characterization of ^{227}Ac in ion-implanted ^{225}Ac or deposited thin film samples for analytic purposes.

References

1. Bernerd, C. *et al.* Production of innovative radionuclides for medical applications at the CERN-MEDICIS facility. *Nucl. Instruments Methods Phys. Res. Sect. B: Beam Interactions with Mater. Atoms* **542**, 137–143 (2023).

2. Johnson, J. D. *et al.* Resonant laser ionization and mass separation of 225Ac. *Sci. Reports* **13**, 1347 (2023).

3. Knoll, G. *Radiation Detection and Measurement*, chap. 3 (Wiley, 2010).

4. Gaiser, J. E. *Charmonium Spectroscopy from Radiative Decays of the J/[psi] and [psi][prime]* (Stanford Linear Accelerator Center, 1982).

5. Morháč, M., Kliman, J., Matoušek, V., Veselskỳ, M. & Turzo, I. Background elimination methods for multidimensional coincidence γ-ray spectra. *Nucl. Instruments Methods Phys. Res. Sect. A: Accel. Spectrometers, Detect. Assoc. Equip.* **401**, 113–132 (1997).

6. Burgess, D. D. & Tervo, R. J. Background estimation for gamma-ray spectrometry. *Nucl. Instruments Methods Phys. Res.* **214**, 431–434 (1983).
